# Decoding Short- and Long-Term Cellular Adaptations to Cr(VI) Exposure Through High-Throughput Transcriptomics

**DOI:** 10.7150/ijms.119668

**Published:** 2026-01-01

**Authors:** I-Jeng Yeh, Chih-Yang Wang, Nam Nhut Phan, Do Thi Minh Xuan, Ching-Chung Ko, Sachin Kumar, Dahlak Daniel Solomon, A-Mei Huang, Meng-Chi Yen, Cheng-Che E. Lan

**Affiliations:** 1 Graduate Institute of Clinical Medicine, College of Medicine, Kaohsiung Medical University, Kaohsiung 80708, Taiwan.; 2 Department of Emergency Medicine, Kaohsiung Medical University Hospital, Kaohsiung Medical University, Kaohsiung 80708, Taiwan.; 3 Graduate Institute of Cancer Biology and Drug Discovery, College of Medical Science and Technology, Taipei Medical University, Taipei 11031, Taiwan.; 4 Ph.D. Program for Cancer Molecular Biology and Drug Discovery, College of Medical Science, Taipei Medical University, Taipei 11031, Taiwan.; 5 TMU Research Center of Cancer Translational Medicine, Taipei Medical University, Taipei 11031, Taiwan.; 6 NTT Institute of Hi-Technology, Nguyen Tat Thanh University, Ho Chi Minh City 70000, Vietnam.; 7 Faculty of Pharmacy, Van Lang University, 69/68 Dang Thuy Tram Street, Binh Loi Trung Ward, Ho Chi Minh City 70000, Vietnam.; 8 Department of Medical Imaging, Chi-Mei Medical Center, Tainan, Taiwan.; 9 Department of Health and Nutrition, Chia Nan University of Pharmacy and Science, Tainan, Taiwan.; 10 School of Medicine, College of Medicine, National Sun Yat-Sen University, Kaohsiung, Taiwan.; 11 Faculty of Applied Sciences and Biotechnology, Shoolini University of Biotechnology and Management Sciences, Himachal Pradesh, 173229, India.; 12 Graduate Institute of Medicine, College of Medicine, Kaohsiung Medical University, Kaohsiung, Taiwan.; 13 Department of Medical Research, Kaohsiung Medical University Hospital, Kaohsiung Medical University, Kaohsiung, Taiwan.; 14 Doctoral Degree Program in Toxicology, College of Pharmacy, Kaohsiung Medical University, Kaohsiung, Taiwan.; 15 Department of Biochemistry, School of Medicine, College of Medicine, Kaohsiung Medical University, Kaohsiung, Taiwan.; 16 Research Center for Environmental Medicine, Kaohsiung Medical University, Kaohsiung, Taiwan.; 17 Department of Dermatology, Kaohsiung Medical University Hospital, Kaohsiung Medical University, Kaohsiung, Taiwan.; 18 Department of Dermatology, College of Medicine, Kaohsiung Medical University, Kaohsiung, Taiwan.

**Keywords:** hexavalent chromium, Cr(VI), transcriptomics, oxidative stress, carcinogenesis, biomarkers

## Abstract

Hexavalent chromium (Cr(VI)) is a well-established environmental and occupational carcinogen, but its time-dependent molecular effects remain poorly characterized. This study aims to elucidate the transcriptional responses triggered by acute versus chronic Cr(VI) exposure through an integrated analysis of two publicly available transcriptomic datasets: GSE16349 (short-term exposure, 16 hours) and GSE24025 (long-term exposure, 4 weeks). We identified 250 differentially expressed genes across both exposure models. MetaCore pathway enrichment analysis revealed shared activation of apoptosis, survival signaling, DNA damage response and repair, and cell cycle progression. Notably, short-term exposure primarily activated acute stress responses, whereas long-term exposure induced reprograms transcription toward fibrosis, EMT, and oncogenic signaling. Protein-protein interaction (PPI) network analysis identified potential key hub genes, with potential as biomarkers for Cr(VI) exposure monitoring. Our findings highlight distinct molecular trajectories in response to Cr(VI) over time, providing valuable insights into the progression from early toxic stress to chronic carcinogenic transformation. These results advance our understanding of Cr(VI)-induced carcinogenesis and suggest these potential targets for preventive and therapeutic interventions in exposed populations.

## 1. Introduction

Chromium (Cr) is an essential trace element in its trivalent form, Cr(III) but is highly toxic and carcinogenic in its hexavalent form Cr(VI) 1, 2. Exposure to Cr(VI) is a well-documented occupational and environmental hazard, leading to severe health risks such as oxidative stress, DNA damage, tumorigenesis, and immune system dysfunction 3, 4. Epidemiological studies have linked chronic Cr(VI) exposure to multiple adverse health outcomes. For instance, dermal exposure to Cr(VI) can cause skin irritation and ulceration 5, while workers in Cr(VI)-related industries experience a significantly higher risk of lung cancer compared with the general population 6. Moreover, ingestion of drinking water or food contaminated with hexavalent chromium poses additional health risks 7. The effects of Cr(VI) on the human body vary widely depending on exposure concentration, duration, and biological context. However, the comprehensive molecular characteristics distinguishing acute from chronic Cr(VI) toxicity remain poorly understood.

High-throughput transcriptomic analyses have provided valuable insights into the molecular pathways underlying Cr(VI)-induced toxicity. For example, Cr(VI) exposure in human dermal fibroblasts has been shown to enrich pathways involved in apoptosis and oxidative stress 8, while exposure of human bronchial epithelial cells promotes carcinogenic transformation through activation of oncogenic pathways 9. In addition, the effects of oral Cr(VI) exposure have been investigated in cell culture as well as in mouse and rat models, revealing systemic toxicity 10, 11. Recent research has also emphasized the roles of histone modifications and non-coding RNAs in Cr(VI)-induced carcinogenesis, highlighting the long-term epigenetic consequences of exposure 12, 13.

Despite extensive research, a direct transcriptomic comparison between short-term and long-term Cr(VI) exposure models has not yet been conducted. This study was designed to address this gap. Because occupational Cr(VI) exposure commonly leads to lung cancer as well as irritation of the skin and airways 14, the present work focused on transcriptomic datasets related to these tissues. Two datasets from the Gene Expression Omnibus (GEO) database were selected for comparative analysis: GSE16349, representing short-term Cr(VI) exposure (16 hours) and profiling gene expression changes in human dermal fibroblasts 8; and GSE24025, representing long-term exposure (4 weeks) and capturing transcriptomic alterations in Cr(VI)-transformed cell colonies 9. This comparative analysis provides new insights into the temporal molecular responses to Cr(VI) toxicity and identifies potential biomarkers for occupational health risk assessment and early detection of Cr(VI)-induced diseases.

## 2. Materials and Methods

### 2.1 Data Collection and Selection Criteria

Two publicly available Gene Expression Omnibus (GEO) datasets from NCBI were selected to investigate the molecular responses to Cr(VI) exposure across different time scales. These datasets were chosen for their well-characterized experimental designs, relevance to Cr(VI) toxicity, and availability of high-quality gene expression profiles. The short-term dataset, GSE16349, contains microarray-based expression profiles from primary human dermal fibroblasts exposed to 5 μM Cr(VI) for 16 hours, capturing acute molecular responses 8. The long-term dataset, GSE24025, includes expression profiles from immortalized epithelial cell colonies (BEAS-2B) subjected to chronic Cr(VI) exposure (0.5 μM for 4 weeks), allowing analysis of transcriptional adaptations under prolonged exposure 9. Raw data were obtained as CEL files (GSE24025) or text-based tables (GSE16349), normalized, and prepared for downstream bioinformatics analysis using methods described in our previous studies 15-17.

### 2.2 Data Preprocessing and Quality Control

For GSE24025, raw CEL files were processed using the Affymetrix package in R. Robust Multi-array Average (RMA) normalization was applied to correct background noise and normalize gene expression intensities. Gene annotation was performed using the hugene10stprobesetSYMBOL function, ensuring accurate probe-to-gene mapping. The GSE16349 dataset, available in a pre-normalized format, was converted into log2-transformed values to maintain consistency across datasets. To evaluate data distribution and detect potential batch effects, boxplots and violin plots were generated for both datasets. Principal Component Analysis (PCA) was performed to assess clustering patterns between control and Cr(VI)-exposed groups. Hierarchical clustering analysis was conducted using the d3heatmap package, allowing visualization of gene expression differences across conditions. The dataset preprocessing and normalization procedures were conducted in R Studio (version 1.2.1335) with R version 4.0.3 following standard bioinformatics workflows 18-20.

### 2.3 Differential Gene Expression Analysis and DEG Selection

Expression matrices from GSE16349 (5 μM Cr(VI), 16 h) and GSE24025 (0.5 μM Cr(VI), 4 weeks) were processed and analyzed independently using the limma package. Probe-level signals were mapped to gene symbols, averaged across probes corresponding to the same gene, and transformed to the log₂ scale. For GSE16349, a two-group comparison was performed between Cr(VI)-exposed and control samples. For GSE24025, comparisons were made between Cr_large versus control and Cr_small versus control groups. Moderated statistics were computed using empirical Bayes shrinkage. Genes were considered differentially expressed if they showed a fold change ≥ 1.2 with a Benjamini-Hochberg-adjusted p < 0.05. In the long-term model, a “shared” gene set was defined as the intersection of the Cr_large and Cr_small DEG lists showing consistent directions of change. “Common DEGs” across exposure durations were defined as genes meeting the differential expression criteria in both datasets with concordant regulation.

### 2.4 Hierarchical Clustering and Principal Component Analysis

To assess sample distribution and transcriptional heterogeneity, hierarchical clustering analysis and PCA were performed. Heatmaps were generated using pheatmap in R to visualize gene expression patterns across control and Cr(VI)-treated groups. PCA plots were constructed using pca3d, allowing for three-dimensional visualization of sample clustering. The separation of Cr(VI)-exposed and control groups provided additional validation of dataset consistency and differential expression trends. The common DEGs were clustered and functionally annotated using the ClusterGVis R package (version 4.5.0) 21. Normalized gene expression values were Z-score transformed and subjected to hierarchical clustering with default parameters to identify groups of genes exhibiting similar expression patterns. The resulting clusters were visualized as a heatmap using the ClusterGVis, which simultaneously displays gene expression profiles, cluster sizes, and average expression trends. Functional enrichment of each cluster was assessed using Gene Ontology Biological Process (GO:BP) and Kyoto Encyclopedia of Genes and Genomes (KEGG) analyses implemented in the clusterProfiler package (version 4.16.0) 22. Enriched terms with adjusted p < 0.05 were considered significant and summarized alongside each gene cluster in the heatmap 23-26.

### 2.5 Pathway Enrichment Analysis

Biological processes associated with gene expression profiles were identified using MetaCore (GeneGo, St. Joseph, MN, USA). Analyses were performed on two gene sets: (i) the DEGs shared by short-term (GSE16349) and long-term (GSE24025) exposure to identify core pathways, and (ii) the DEGs specific to each dataset to capture duration-dependent biology 27-30. Statistical significance was assessed with two-tailed Fisher's exact tests (p < 0.05).

### 2.6 Protein-Protein Interaction (PPI) Network Analysis

To further elucidate the Cr(VI)-responsive molecular interaction networks, Protein-Protein Interaction (PPI) analysis was conducted using the STRING database (version 12.0, https://string-db.org) 31. A total of 250 DEGs were uploaded to STRING, with *Homo sapiens* selected as the reference organism. The minimum required interaction score was set to 0.7 (high confidence), and active interaction sources included experimental evidence, co-expression, and curated database annotations. K-means clustering (k = 10) was applied to partition the network into functionally distinct modules. The resulting interaction network was visualized in Cytoscape software (version 3.10.4) 32. Network topological analysis was subsequently conducted using the cytoHubba plugin to identify hub genes based on three centrality measures: (1) degree centrality, representing the number of direct interactions; (2) betweenness centrality, indicating a node's control over information flow within the network; and (3) closeness centrality. Genes ranking within the top 10 for each measure were defined as hub genes 33-36.

## 3. Results

### 3.1 Differential Gene Expression Analysis Reveals Common and Distinct Responses to Short-term and Long-term Cr(VI) Exposure

**Figure 1:**
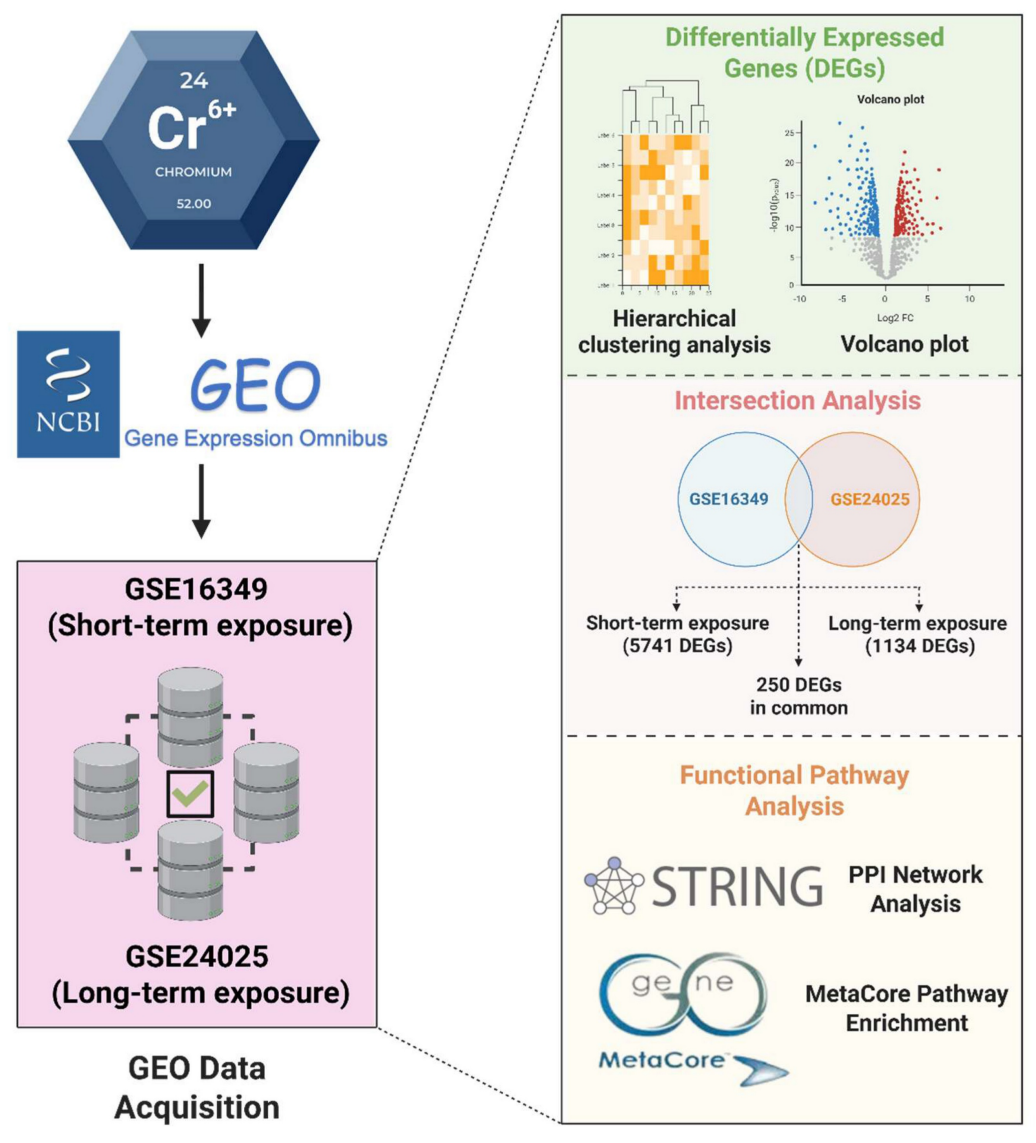
** Overview of the study workflow.** Short-term (GSE16349, 16 h) and long-term (GSE24025, 4 weeks) Cr(VI) exposure datasets were independently analyzed to identify DEGs. The intersection analysis defined shared DEGs and those unique to each exposure duration. Functional and network analyses were conducted using STRING for protein-protein interactions and MetaCore for pathway enrichment.

**Figure 2:**
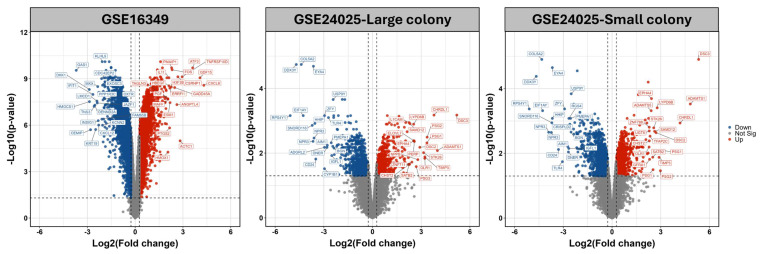
** Volcano plots comparing chromium (VI)-exposed samples to matched controls.** (A) GSE16349; (B) GSE24025-Large colony; (C) GSE24025-Small colony. Points are colored by significance: red, up-regulated; blue, down-regulated; grey, not significant. Vertical dashed lines mark |Fold change| = 1.2, and the horizontal dashed line marks p-value = 0.05). Top 20 DEGs are labeled.

The analysis workflow of this study is illustrated in Figure 1. To systematically compare transcriptomic alterations induced by short-term (16 h) and long-term (4-week) Cr(VI) exposure, we identified differentially expressed genes (DEGs) defined by fold change (FC) ≥ 1.2 and Benjamini-Hochberg-adjusted p < 0.05. In the short-term dataset (GSE16349), 5741 DEGs were detected (2825 upregulated; 2916 downregulated). In the long-term model (GSE24025), comparisons of Cr_large and Cr_small colonies versus untreated controls yielded 1590 and 2359 DEGs, respectively (Figure 2). Principal component analysis (PCA) revealed that Cr_large and Cr_small colonies exhibited highly similar transcriptional profiles (Supplementary Figure S1), which was further supported by hierarchical clustering analysis showing consistent gene expression patterns between the two groups (Supplementary Figure S2). Intersection analysis identified 1134 shared DEGs (455 upregulated; 679 downregulated) between Cr_large and Cr_small colonies, justifying their combination into a unified long-term Cr(VI)-treated group for downstream analyses. To identify genes commonly responsive to Cr(VI) exposure across different durations, the 1134 shared DEGs from the long-term model were compared with those from the short-term dataset. This comparison revealed 86 genes consistently upregulated and 164 consistently downregulated, defining a core Cr(VI)-responsive signature conserved between acute and chronic exposure conditions.

**Figure 3:**
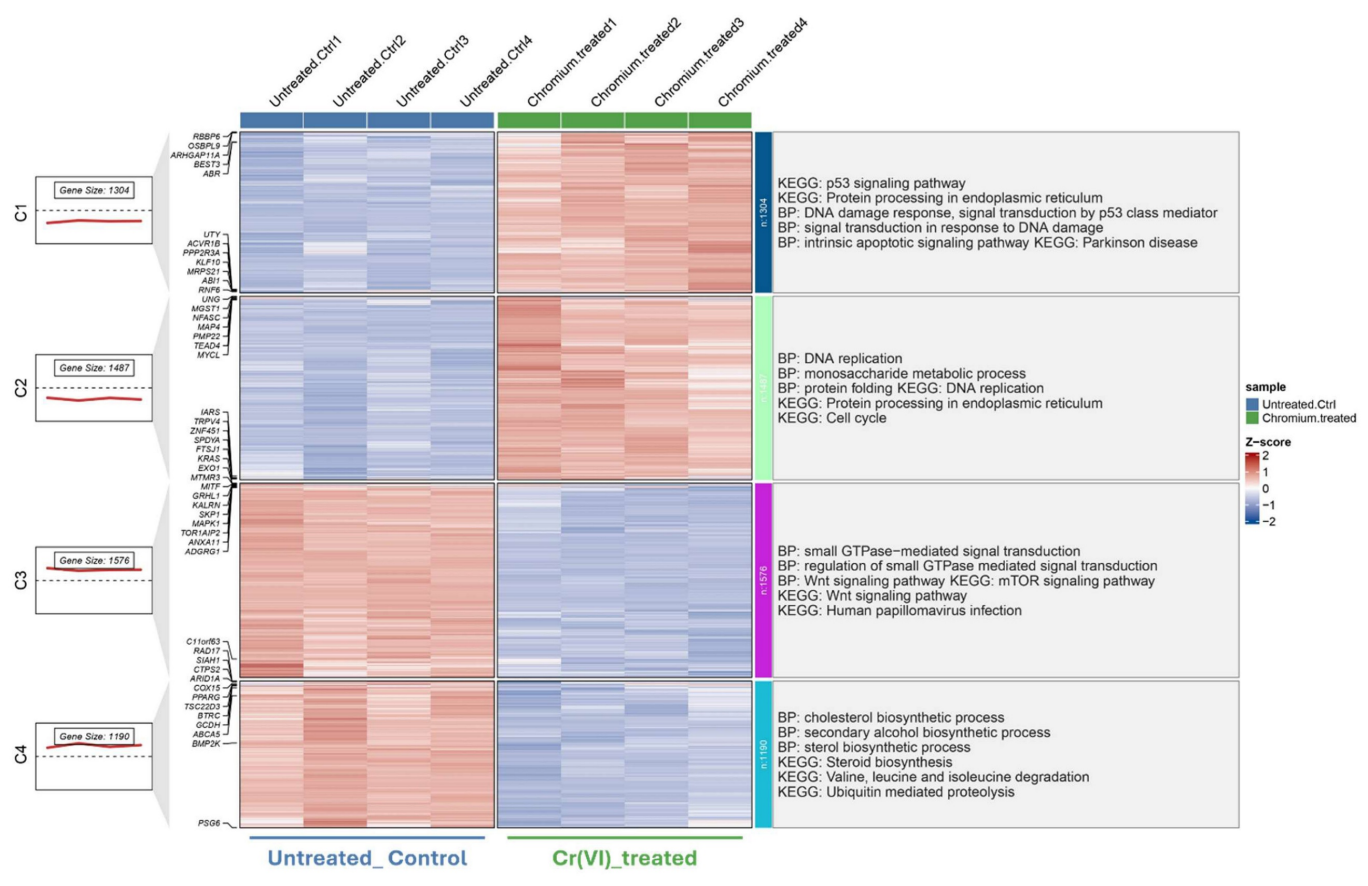
** Transcriptomic clustering and functional enrichment of genes altered by Cr(VI) exposure (GSE16349)**. Hierarchical clustering of DEGs identified from GSE16349, comparing untreated control cells and Cr(VI)-treated cells. Each column represents an individual sample, and each row represents a gene. Expression values are shown as Z-scores, with red indicating upregulation and blue indicating downregulation relative to the mean expression. The left panels show the number of genes and average expression trend within each cluster. Functional enrichment analyses (Gene Ontology Biological Process and KEGG pathways) for each cluster are summarized on the right.

To further explore distinct molecular responses under acute and chronic Cr(VI) exposure, clusterGVis analysis was performed to visualize gene clusters derived from GO and KEGG enrichment results. Specifically, 5741 DEGs from the short-term exposure dataset (GSE16349) and 1134 DEGs from the long-term model (GSE24025) were subjected to functional enrichment and cluster visualization analyses, respectively, using the ClusterGVis package. In the short-term model (Figure 3), ClusterGVis identified four major gene clusters with distinct transcriptional patterns. Genes in clusters 1 and 2 were upregulated and enriched in biological processes related to DNA damage response, p53 signaling, and protein processing in the endoplasmic reticulum, reflecting acute stress responses. In contrast, clusters 3 and 4 were downregulated and primarily associated with cholesterol biosynthesis, metabolic regulation, and cell cycle progression.

**Figure 4:**
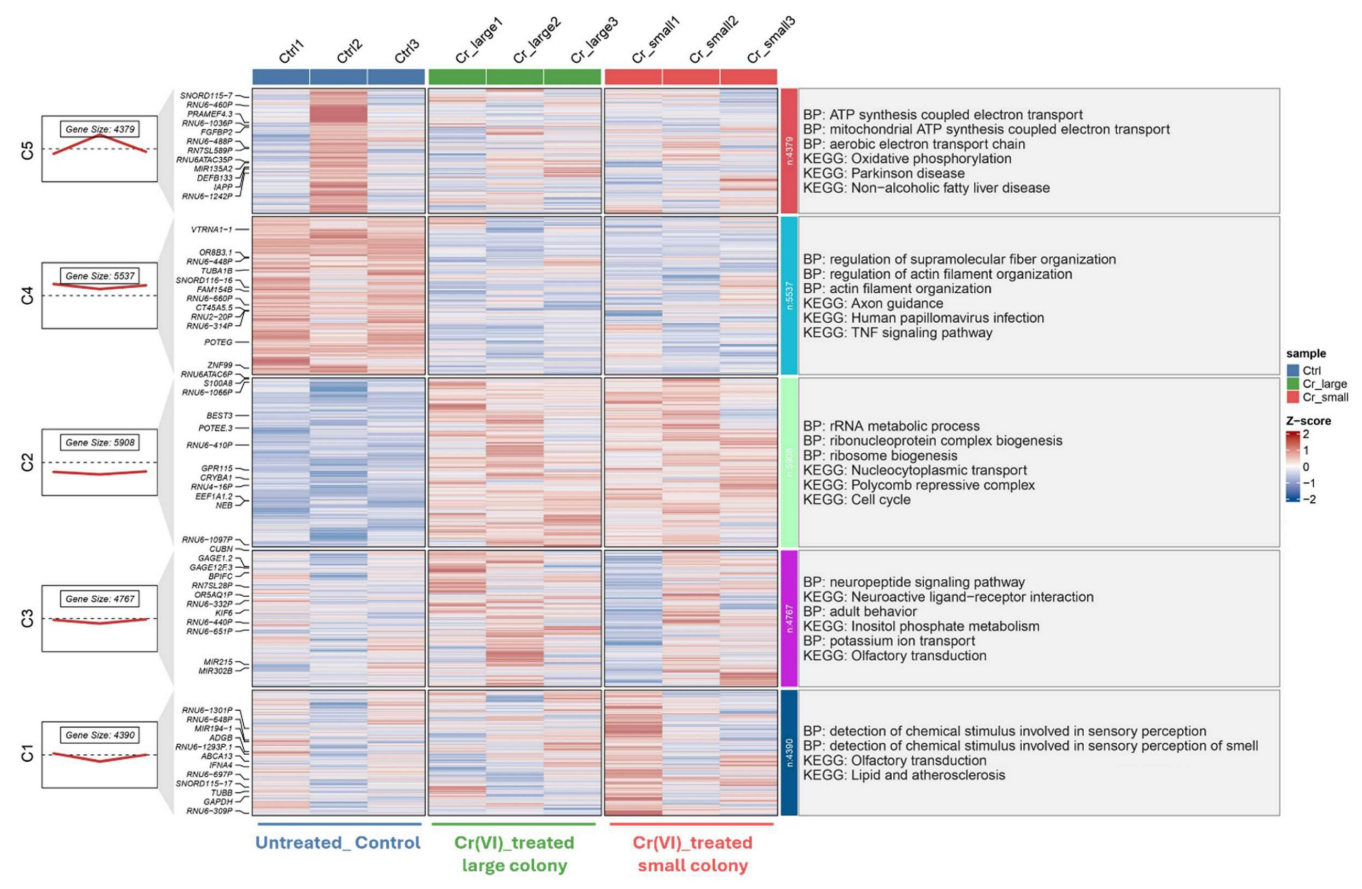
** Transcriptomic clustering and functional enrichment of genes altered by Cr(VI) exposure (GSE24025).** Hierarchical clustering of DEGs from dataset GSE24025, comparing untreated control (Ctrl), Cr(VI)-treated large colony, and Cr(VI)-treated small colony groups. Each column represents an individual sample, and each row represents a gene. Expression values are shown as Z-scores, with red indicating upregulation and blue indicating downregulation relative to the mean expression. The left panels show the number of genes and average expression trend within each cluster. Functional enrichment analyses (Gene Ontology Biological Process and KEGG pathways) for each cluster are summarized on the right.

In the long-term model (Figure 4), five gene clusters were identified, highlighting persistent transcriptional reprogramming under prolonged Cr(VI) exposure. Upregulated clusters were significantly enriched in mitochondrial ATP synthesis, rRNA metabolic processes, and actin filament organization, whereas downregulated clusters were associated with neuroactive ligand-receptor interaction and lipid metabolism. Collectively, these results indicate a shift from transient stress-related responses during short-term exposure to sustained metabolic and structural adaptation following chronic Cr(VI) treatment.

### 3.2 Functional Pathway Enrichment Highlights Distinct Short-term and Long-term Cr(VI) Toxicity Mechanisms

**Figure 5:**
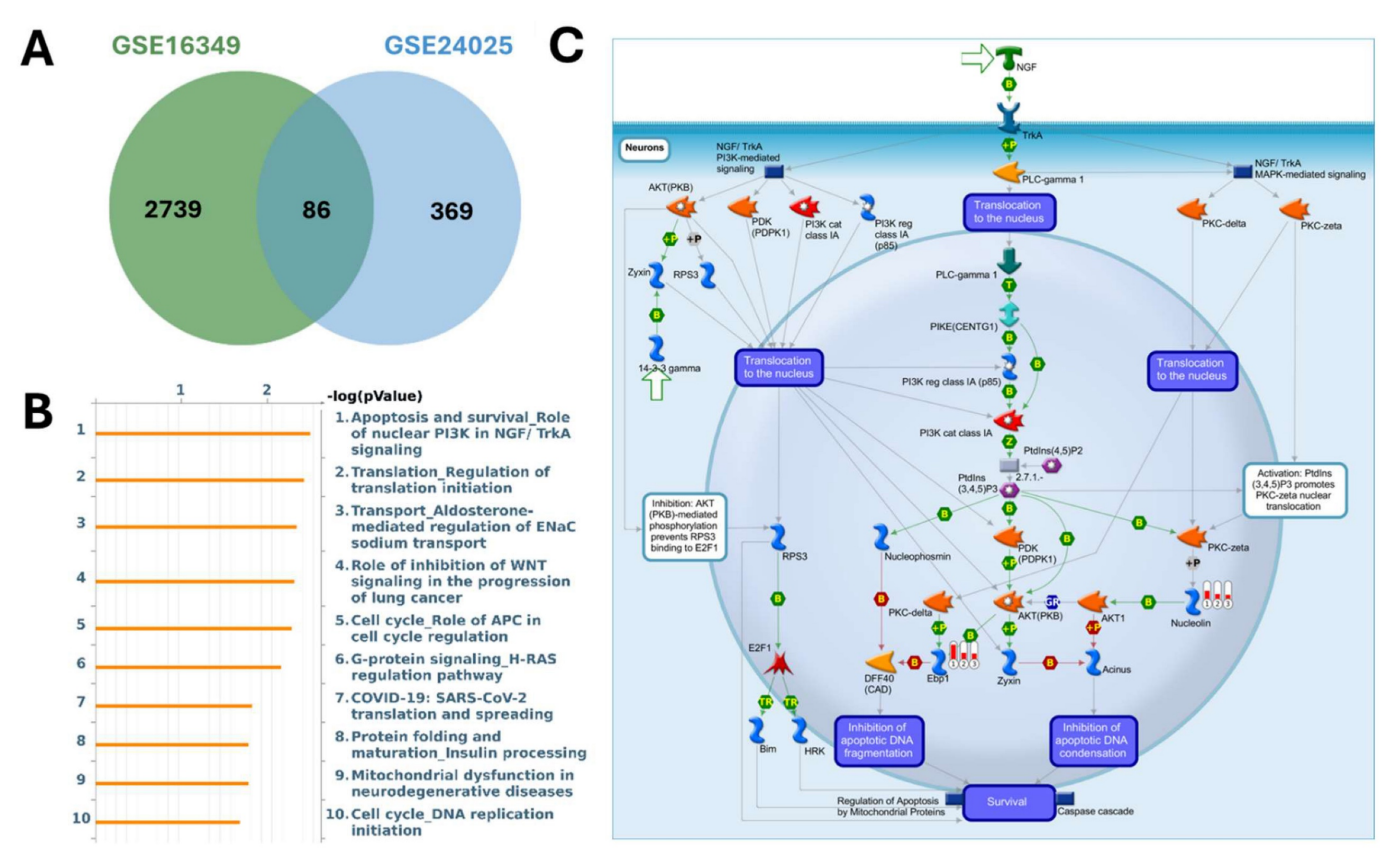
** MetaCore pathway analysis of pathways regulated by the shared up-regulated genes from GSE16349 and GSE24025 (chromium-exposed vs control).** (A) Venn diagram showing 2739 up-regulated genes unique to GSE16349, 369 genes unique to GSE24025, and 86 up-regulated genes in both datasets. (B) Top 10 enriched MetaCore pathways for the shared up-regulated set ranked by -log10(p-value). (C) MetaCore process map for the top pathway, “Apoptosis and survival-Role of nuclear PI3K in NGF/TrkA signaling,” with the shared up-regulated genes overlaid, highlighting the PI3K/AKT-mediated survival module. Up-regulation was defined in each dataset as log2(FC) ≥ 1.2 with p-value < 0.05.

**Figure 6:**
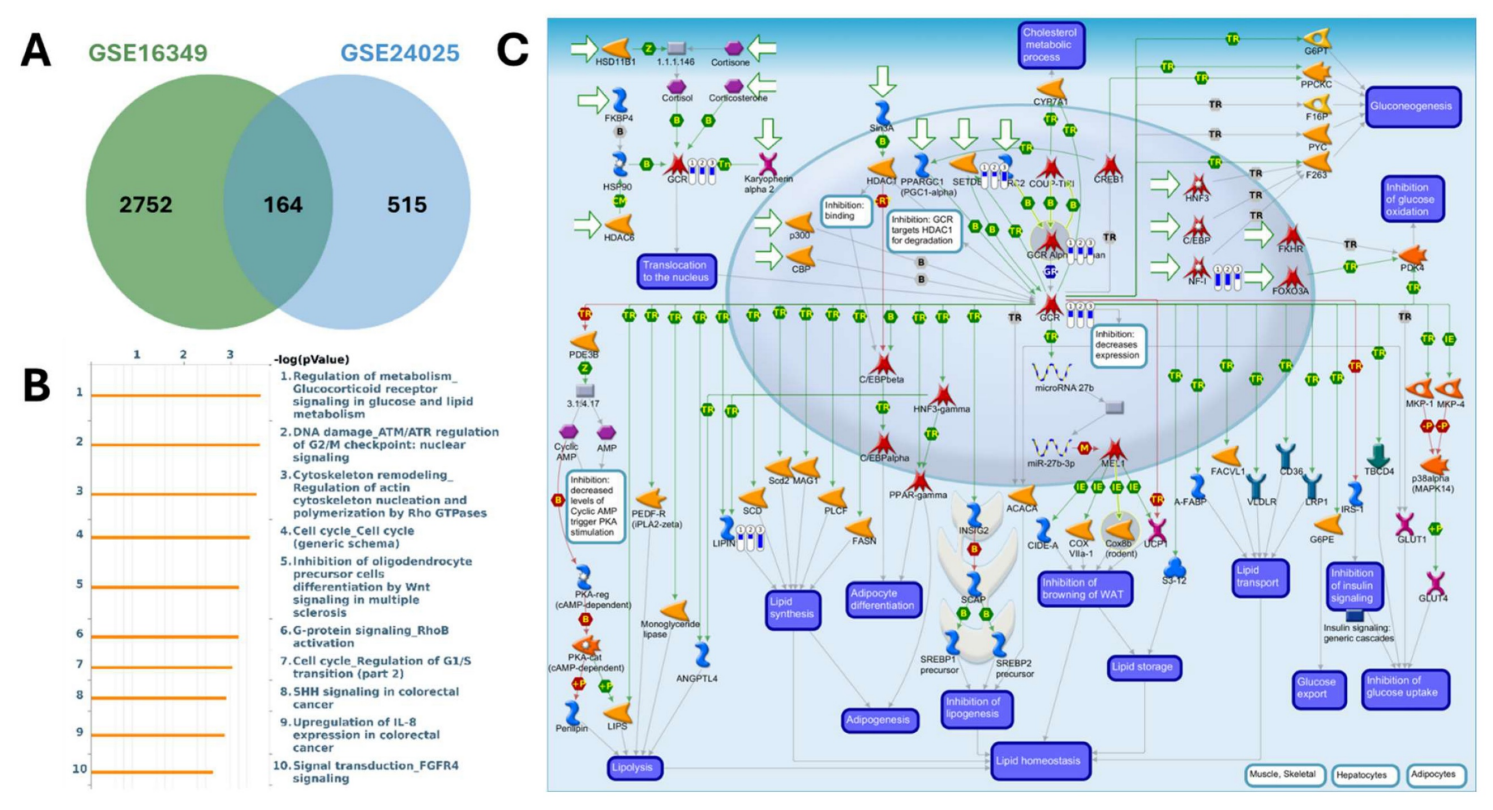
** MetaCore pathway analysis of pathways regulated by the shared down-regulated genes from GSE16349 and GSE24025 (chromium-exposed vs control).** (A) Venn diagram showing 2752 down-regulated genes unique to GSE16349, 515 genes unique to GSE24025, and 164 down-regulated genes in both datasets. (B) Top 10 enriched MetaCore pathways for the shared down-regulated set ranked by -log10(p-value); (C) MetaCore process map for the top pathway, “Regulation of metabolism-Glucocorticoid receptor signaling in glucose and lipid metabolism,” with the shared down-regulated genes mapped, highlighting modules governing lipolysis, lipogenesis, adipogenesis, insulin signaling, and lipid homeostasis. Down-regulation was defined as log2(FC) ≤ -1.2 with p-value < 0.05.

MetaCore pathway enrichment analysis was applied to the common DEGs to identify core pathways activated under both short- and long-term exposure. Among the upregulated genes, the top enriched pathways were largely associated with apoptosis and survival signaling (nuclear PI3K/NGF-TrkA signaling, p53 activation, and APC-regulated cell cycle control), DNA damage response and repair (DNA replication initiation, mismatch repair, and apoptosis regulation), and cell cycle progression (initiation of mitosis and metaphase checkpoint). Additional categories included G-protein-mediated signaling (H-RAS, K-RAS, N-RAS, and Gα subunits), protein folding and maturation (insulin processing, CFTR regulation), and immune-related signaling (Treg regulation in COPD, IL-6 signaling in adipocytes, and CD4^+^ T-cell memory generation). Collectively, these findings suggest that Cr(VI) exposure activates transcriptional programs promoting cell survival, stress adaptation, metabolic regulation, and immune modulation, consistent with pathways that support immune suppression and carcinogenic transformation (Figure 5, Supplementary Table S1). In contrast, the downregulated genes were enriched in pathways tied to cell cycle regulation and DNA damage checkpoints, such as ATM/ATR-mediated G2/M control, G1/S progression, prometaphase chromosome condensation, and homologous recombination repair. Suppression was also observed in WNT/β-catenin and TGF-β signaling (linked to hepatocellular carcinoma, lung cancer, and pancreatic cancer), pointing to impaired regulation of tissue homeostasis and tumor-suppressive signaling. Additional enrichment involved immune and inflammatory processes (IL-8 signaling, IFN-α/β-MAPK signaling, M-CSF receptor signaling, and Th17 differentiation) as well as fibrosis/ECM remodeling (stellate cell activation, Angiotensin II-PI3K/ERK signaling, and EMT regulation). Together, these results indicate that Cr(VI) exposure is associated with downregulation of genomic maintenance, checkpoint control, and immune surveillance, thereby fostering genomic instability, immune evasion, and a tumor-permissive microenvironment (Figure 6, Supplementary Table S2).

To further distinguish exposure-specific transcriptional responses, pathway enrichment analyses were performed on distinct DEGs from the acute (GSE16349) and chronic (GSE24025) models. In the acute exposure dataset (GSE16349), enriched pathways highlighted rapid activation of oxidative stress and hypoxia responses (ROS signaling, HIF-1 transcriptional targets, and negative regulation of HIF1A function). Robust engagement of DNA damage response and repair was also evident (ATM/ATR activation, p53 signaling, and DNA replication initiation/elongation), together with apoptosis and survival pathways (p53- and p73-dependent apoptosis, PDGF-PI3K/AKT-NFκB, and mTORC1 signaling). Additional enriched categories included cytoskeletal remodeling and motility (Rho GTPase-regulated actin organization, S1P1 receptor signaling, PDGF-Rho GTPase signaling), cell cycle checkpoints (Cullin1/Rbx1 E3 ligase control, G1/S transition, mitotic control), and oncogenic/developmental signaling (WNT/β-catenin, NOTCH, TGF-β, and EGFR). Immune-related and pro-fibrotic signaling (IL-3, IL-8, BCR, Th2/TNF-α-induced fibrosis, stellate cell activation) were also enriched. These results suggest that short-term Cr(VI) exposure triggers an immediate stress-response program characterized by ROS production, DNA damage repair, and apoptosis regulation, while simultaneously perturbing immune, fibrotic, and developmental pathways. Such responses may serve as adaptive mechanisms to genotoxic stress but also create vulnerabilities that predispose carcinogenesis (Supplementary Figure S3, Supplementary Table S3). By contrast, the chronic exposure dataset (GSE24025) revealed sustained enrichment of fibrotic and pro-tumorigenic signaling pathways. These included stellate cell activation and liver fibrosis, multiple TGF-β-driven programs (stimulation in lung, breast, pancreatic, and colorectal cancers; SMAD signaling; Activin A signaling; and EMT induction), and EGFR- and WNT/β-catenin-mediated transcriptional regulation. Chronic exposure also impacted cell cycle control, with enrichment of G1/S regulation, APC- and Cul1/Rbx1-mediated checkpoints, senescence, and metaphase transition. In addition, immune and inflammation-related pathways were prominent, including IL-6 signaling (in breast and prostate cancer) and Hedgehog/IGF/HGF cooperation in stem cell regulation, suggesting acquisition of cancer stemness traits. ECM remodeling and fibrosis-related signatures (fibroblast/myofibroblast activation, systemic sclerosis, and metalloprotease signaling) further underscore the emergence of a fibrotic, tumor-permissive microenvironment (Supplementary Figure S4, Supplementary Table S4).

In summary, short-term exposure is dominated by acute stress responses (oxidative stress, DNA damage repair, and apoptosis), whereas long-term exposure reprograms transcription toward fibrosis, EMT, and oncogenic signaling. Together, these findings capture a continuum from immediate adaptation to chronic carcinogenic transformation.

### 3.3 Protein-Protein Interaction Network Identifies Key Hub Genes in Cr(VI) Exposure

**Figure 7:**
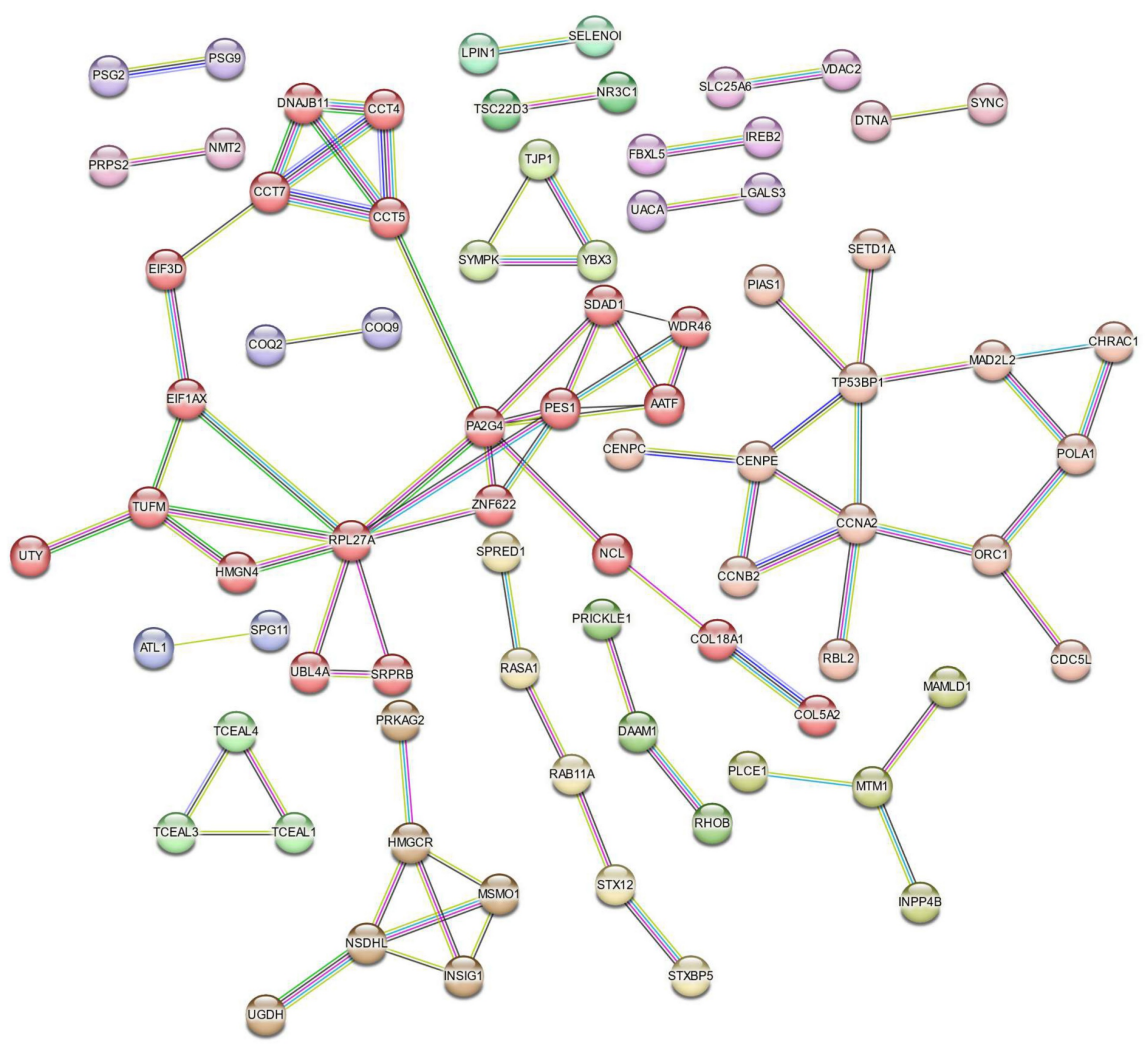
** STRING-based Protein-Protein Interaction (PPI) network of Cr(VI)-responsive genes.** The network consists of 84 edges (interactions) among 250 nodes (proteins) derived from Cr(VI)-responsive DEGs. A high-confidence interaction score (≥ 0.7) was applied, and nodes are color-coded by k-means clustering (k = 18). Only connected nodes are displayed.

Protein-protein interaction (PPI) analysis using the STRING database revealed a network comprising 250 nodes and 84 edges, indicating extensive interconnectivity among the DEGs (Figure 7). K-means clustering (k = 18) further partitioned the network into distinct submodules, many of which were enriched in ribosome biogenesis and protein folding, cell-cycle regulation, sterol biosynthesis, phosphatidylinositol phosphate metabolism, TGF-β activation, ubiquinol biosynthesis, and apoptosis-related pathways. The largest cluster contained AATF, CCT4, CCT5, CCT7, EIF3D, and HMGN4, representing a tightly connected chaperonin-ribosomal module central to proteostatic control, while smaller clusters captured specialized metabolic and signaling processes such as LGALS3-mediated apoptosis and ferritin complex formation, underscoring the multifaceted cellular response to Cr(VI).

**Figure 8:**
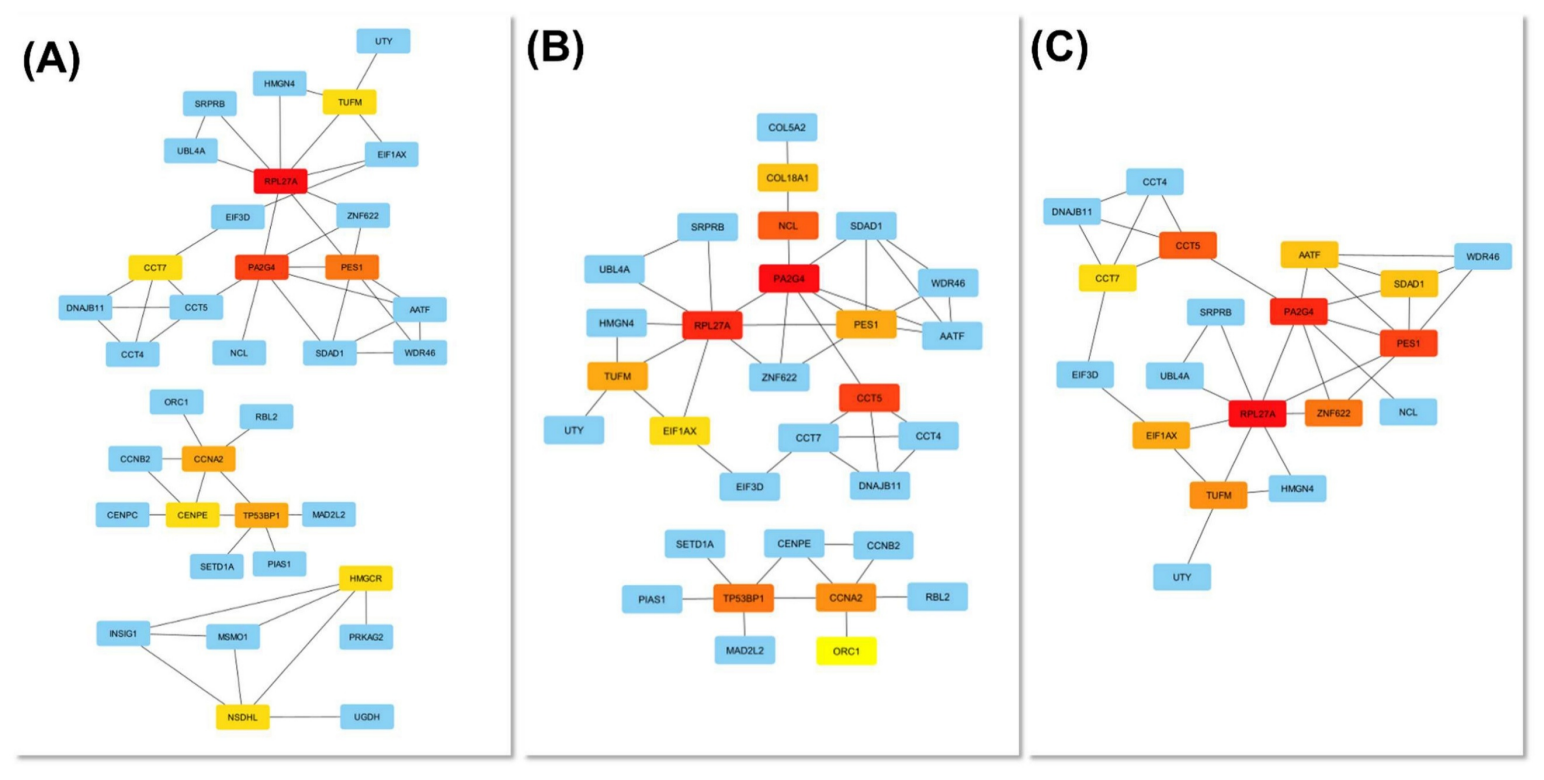
** Identification of hub genes in the Cr(VI)-responsive PPI network.** Hub genes were determined using three cytoHubba centrality metrics: (A) Degree, (B) Betweenness, and (C) Closeness. The top 10 ranked nodes for each metric are visualized in the corresponding subnetworks. Node color intensity represents hub ranking (red = higher centrality). Highly ranked nodes, particularly PL27A, PA2G4, and PES, formed a densely interconnected module enriched in ribosomal and nucleolar proteins, highlighting a nucleolar-ribosome-biogenesis axis as the topological core of the network.

To identify key molecular mediators within this network, topological analysis was performed using the cytoHubba plugin in Cytoscape. Three centrality metrics, including degree, betweenness, and closeness, were applied to evaluate node importance from complementary perspectives (Figure 8A-C). Across all three analyses, RPL27A, PA2G4, and PES1 consistently emerged as core hubs, underscoring their pivotal roles in maintaining network connectivity and mediating adaptation to chromium exposure. In contrast, CCT5, CCT7, and EIF3D ranked highly in two of the three analyses, reflecting strong but context-dependent participation in proteostasis and chaperonin-assisted folding. The degree and closeness networks were largely concordant, highlighting a compact nucleolar- translational core composed of RPL27A, PA2G4, PES1, and EIF3D, together with a CCT chaperonin mini-cluster (CCT5, CCT7). The betweenness map preserved this structure but additionally elevated AATF, SDAD1, and WDR46 as bridging nodes linking RNA-processing and chaperone/cell-cycle modules.

When the top-ranked nodes from all three centrality metrics were integrated, six consensus hub genes, including RPL27A, PA2G4, PES1, CCT5, CCT7, and EIF3D, were identified, representing genes that combine extensive interactions (high degree and closeness) with critical information flow across functional submodules (high betweenness). Collectively, these findings define a tightly integrated PPI network organized around two principal functional axes: ribosome biogenesis and translation initiation (RPL27A, PA2G4, PES1, EIF3D) and proteostasis via the CCT foldase complex (CCT5, CCT7), underscoring the nucleolar-ribosomal machinery as a central element of the cellular response to Cr(VI)-induced stress.

## 4. Discussion

**Figure 9:**
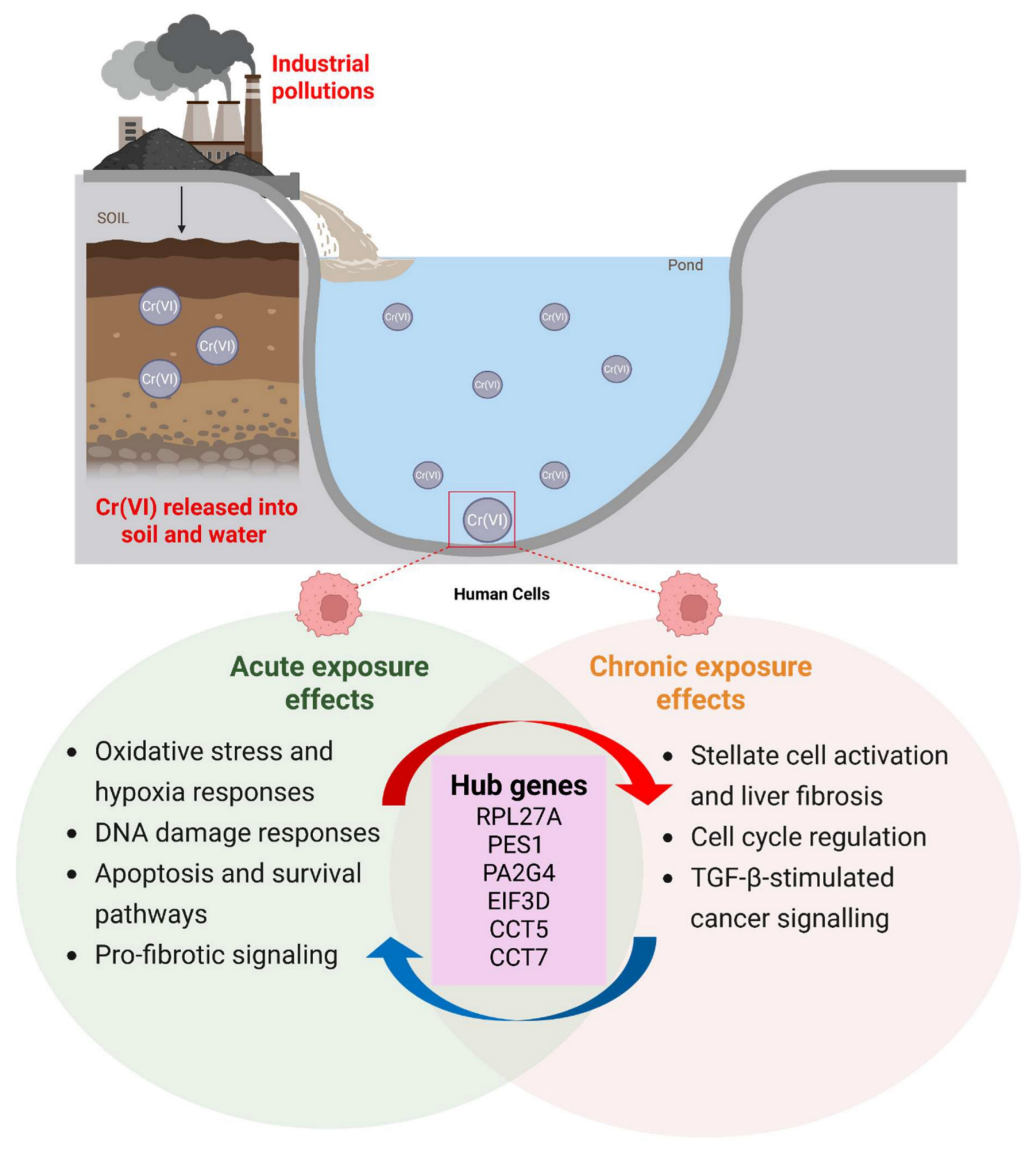
Proposed model summarizing the molecular effects of acute and chronic hexavalent chromium (Cr(VI)) exposure.

This study provides a comprehensive comparative transcriptomic analysis of short-term and long-term Cr(VI) exposure, revealing both shared and time-dependent molecular alterations. By integrating two independent GEO datasets representing acute (GSE16349, 16 hours) and chronic (GSE24025, 4 weeks) Cr(VI) exposure, we identified 250 common DEGs and uncovered distinct transcriptional programs underlying the transition from immediate stress responses to chronic carcinogenic transformation. A schematic summary of the findings is illustrated in Figure 9.

Our findings demonstrate that short-term Cr(VI) exposure primarily triggers acute stress responses characterized by activation of oxidative stress, DNA damage repair, and apoptotic pathways. This observation aligns with prior studies showing that Cr(VI) rapidly generates reactive oxygen species (ROS) and induces genotoxic stress, activating p53-dependent and p73-mediated apoptosis signaling 37-41. The early upregulation of DNA repair and checkpoint genes observed here likely reflects a protective response aimed at maintaining genomic stability under transient oxidative insult. In contrast, prolonged exposure to Cr(VI) led to extensive transcriptional reprogramming associated with fibrosis, epithelial-mesenchymal transition (EMT), and oncogenic signaling. Enrichment of TGF-β, WNT/β-catenin, and EGFR pathways in the chronic model suggests a shift toward pro-tumorigenic and pro-fibrotic signaling, consistent with the progression from adaptive to maladaptive responses. These results are consistent with previous findings that long-term Cr(VI) exposure promotes EMT, epigenetic alterations, and anchorage-independent growth in human bronchial epithelial cells 42-44. Together, these observations suggest that sustained Cr(VI) exposure drives a temporal continuum of molecular events, from acute genotoxic stress to chronic transcriptional remodeling, culminating in carcinogenic transformation.

PPI network analysis highlighted a ribosomal-centered network dominated by RPL family members (RPL27A), along with nucleolar proteins such as PES1 and PA2G4. These hub genes are well-known regulators of ribosome biogenesis, translational control, and cell cycle progression. Notably, RPL5 and RPL11 form part of the 5S ribonucleoprotein complex that modulates p53 activity by sequestering MDM2, thereby linking ribosome biogenesis stress to genome surveillance 45, 46. Their upregulation in both acute and chronic exposure models suggests persistent activation of nucleolar stress signaling. The concurrent enrichment of rRNA processing and mitochondrial translation pathways further supports the notion that Cr(VI) toxicity disrupts proteostasis and energy metabolism. Given that dysregulated ribosome biogenesis is increasingly recognized as a hallmark of cancer, the dominance of ribosomal proteins within the Cr(VI) PPI network underscores their potential role in driving chronic toxicity and malignant transformation 46-48. Integrating functional enrichment results across both exposure models suggests a temporal shift in cellular priorities. During acute exposure, cells mount a coordinated antioxidant, DNA repair, and apoptotic response aimed at mitigating damage. However, under sustained exposure, these defensive mechanisms give way to pathways promoting cell proliferation, ECM remodeling, and immune modulation. Suppression of tumor-suppressive signaling (WNT/β-catenin and TGF-β regulation) and dysregulation of immune pathways (IL-6, IFN-α/β, and Th17 signaling) were observed, collectively favoring a microenvironment conducive to malignant transformation. These transcriptional transitions reflect the biological trajectory from oxidative stress-driven cytotoxicity to fibrotic remodeling and eventual neoplastic progression, consistent with epidemiological evidence linking chronic Cr(VI) exposure to lung carcinogenesis 49, 50.

The identification of common DEGs and hub genes across both exposure durations provides a foundation for developing biomarkers to monitor Cr(VI) exposure and early cellular transformation. In particular, RPL27A and PES1 may serve as sentinel indicators of Cr(VI)-induced nucleolar stress, while fibrosis-related genes involved in ECM remodeling and TGF-β signaling could mark chronic adaptation and disease progression. Validation of these genes in exposed human cohorts or experimental animal models would strengthen their translational potential for occupational health surveillance.

Despite its integrative scope, this study has several limitations. First, the analysis relied on pre-existing datasets derived from distinct cell types and experimental conditions, which may introduce confounding biological variability. Second, transcriptomic profiling captures only mRNA-level changes, while Cr(VI) toxicity also involves post-translational and epigenetic mechanisms not addressed here. Future studies combining transcriptomics with proteomic and epigenomic analyses will be essential to fully delineate the multilayered response to Cr(VI). Experimental validation of candidate biomarkers and hub genes in human tissues and *in vivo* models will further clarify their functional relevance and diagnostic utility.

## 5. Conclusion

In conclusion, this study provides a comprehensive comparison of transcriptomic responses to short-term and long-term hexavalent chromium exposure. Acute exposure primarily activated oxidative stress, DNA repair, and apoptotic pathways, whereas prolonged exposure promoted transcriptional reprogramming associated with fibrosis, epithelial-mesenchymal transition, and oncogenic signaling. The consistent involvement of nucleolar and ribosomal proteins highlights their central role in stress adaptation and cellular transformation. Together, these findings offer new insights into the molecular progression from early toxic injury to chronic carcinogenesis and identify potential biomarkers for monitoring and mitigating Cr(VI)-induced health risks.

## Supplementary Material

Supplementary figures and table.

## References

[1] Association ICD (2007). Health Safety and Environment Guidelines for Chromium. Revision.

[2] Association ICD (1997). Criteria document for hexavalent chromium. Paris.

[3] Kart A, Koc E, Dalginli KY, Gulmez C, Sertcelik M, Atakisi O (2016). The therapeutic role of glutathione in oxidative stress and oxidative DNA damage caused by hexavalent chromium. Biological trace element research.

[4] Patlolla AK, Barnes C, Yedjou C, Velma V, Tchounwou PB (2009). Oxidative stress, DNA damage, and antioxidant enzyme activity induced by hexavalent chromium in Sprague-Dawley rats. Environmental Toxicology: An International Journal.

[5] Shin DY, Lee SM, Jang Y, Lee J, Lee CM, Cho EM (2023). Adverse Human Health Effects of Chromium by Exposure Route: A Comprehensive Review Based on Toxicogenomic Approach. Int J Mol Sci.

[6] Proctor DM, Bhat V, Suh M, Reichert H, Jiang X, Thompson CM (2021). Inhalation cancer risk assessment for environmental exposure to hexavalent chromium: Comparison of margin-of-exposure and linear extrapolation approaches. Regul Toxicol Pharmacol.

[7] Reif BM, Murray BP (2025). Chromium Toxicity. StatPearls. Treasure Island (FL) ineligible companies. Disclosure: Brian Murray declares no relevant financial relationships with ineligible companies.

[8] Sellamuthu R, Umbright C, Chapman R, Leonard S, Li S, Kashon M (2011). Transcriptomics evaluation of hexavalent chromium toxicity in human dermal fibroblasts. J Carcinog Mutagen.

[9] Sun H, Clancy HA, Kluz T, Zavadil J, Costa M (2011). Comparison of gene expression profiles in chromate transformed BEAS-2B cells. PloS one.

[10] Thompson CM, Rager JE, Suh M, Ring CL, Proctor DM, Haws LC (2016). Transcriptomic responses in the oral cavity of F344 rats and B6C3F1 mice following exposure to Cr(VI): Implications for risk assessment. Environ Mol Mutagen.

[11] Rager JE, Ring CL, Fry RC, Suh M, Proctor DM, Haws LC (2017). High-Throughput Screening Data Interpretation in the Context of In Vivo Transcriptomic Responses to Oral Cr(VI) Exposure. Toxicol Sci.

[12] Arita A, Costa M (2009). Epigenetics in metal carcinogenesis: nickel, arsenic, chromium and cadmium. Metallomics.

[13] Iyer M, Anand U, Thiruvenkataswamy S, Babu HWS, Narayanasamy A, Prajapati VK (2023). A review of chromium (Cr) epigenetic toxicity and health hazards. Science of the Total Environment.

[14] Verdonck J, Duca RC, Galea KS, Iavicoli I, Poels K, Toreyin ZN (2021). Systematic review of biomonitoring data on occupational exposure to hexavalent chromium. Int J Hyg Environ Health.

[15] Chen PS, Hsu HP, Phan NN, Yen MC, Chen FW, Liu YW (2021). CCDC167 as a potential therapeutic target and regulator of cell cycle-related networks in breast cancer. Aging (Albany NY).

[16] Wu YH, Yeh IJ, Phan NN, Yen MC, Liu HL, Wang CY (2021). Severe acute respiratory syndrome coronavirus (SARS-CoV)-2 infection induces dysregulation of immunity: in silico gene expression analysis. Int J Med Sci.

[17] Cheng LC, Kao TJ, Phan NN, Chiao CC, Yen MC, Chen CF (2021). Novel signaling pathways regulate SARS-CoV and SARS-CoV-2 infectious disease. Medicine (Baltimore).

[18] Wu CC, Ekanem TI, Phan NN, Loan DTT, Hou SY, Lee KH (2020). Gene signatures and prognostic analyses of the Tob/BTG pituitary tumor-transforming gene (PTTG) family in clinical breast cancer patients. Int J Med Sci.

[19] Liu HL, Yeh IJ, Phan NN, Wu YH, Yen MC, Hung JH (2020). Gene signatures of SARS-CoV/SARS-CoV-2-infected ferret lungs in short- and long-term models. Infect Genet Evol.

[20] Wang CY, Chao YJ, Chen YL, Wang TW, Phan NN, Hsu HP (2021). Upregulation of peroxisome proliferator-activated receptor-α and the lipid metabolism pathway promotes carcinogenesis of ampullary cancer. Int J Med Sci.

[21] Kumar L, M EF (2007). Mfuzz: a software package for soft clustering of microarray data. Bioinformation.

[22] Yu G, Wang LG, Han Y, He QY (2012). clusterProfiler: an R package for comparing biological themes among gene clusters. Omics.

[23] Kao TJ, Wu CC, Phan NN, Liu YH, Ta HDK, Anuraga G (2021). Prognoses and genomic analyses of proteasome 26S subunit, ATPase (PSMC) family genes in clinical breast cancer. Aging (Albany NY).

[24] Wu YH, Yeh IJ, Phan NN, Yen MC, Hung JH, Chiao CC (2021). Gene signatures and potential therapeutic targets of Middle East respiratory syndrome coronavirus (MERS-CoV)-infected human lung adenocarcinoma epithelial cells. J Microbiol Immunol Infect.

[25] Li CY, Anuraga G, Chang CP, Weng TY, Hsu HP, Ta HDK (2023). Repurposing nitric oxide donating drugs in cancer therapy through immune modulation. J Exp Clin Cancer Res.

[26] Xuan DTM, Yeh IJ, Wu CC, Su CY, Liu HL, Chiao CC (2022). Comparison of Transcriptomic Signatures between Monkeypox-Infected Monkey and Human Cell Lines. J Immunol Res.

[27] Xuan DTM, Yeh IJ, Liu HL, Su CY, Ko CC, Ta HDK (2025). A comparative analysis of Marburg virus-infected bat and human models from public high-throughput sequencing data. Int J Med Sci.

[28] Anuraga G, Lang J, Xuan DTM, Ta HDK, Jiang JZ, Sun Z (2024). Integrated bioinformatics approaches to investigate alterations in transcriptomic profiles of monkeypox infected human cell line model. J Infect Public Health.

[29] Xuan DTM, Yeh IJ, Su CY, Liu HL, Ta HDK, Anuraga G (2023). Prognostic and Immune Infiltration Value of Proteasome Assembly Chaperone (PSMG) Family Genes in Lung Adenocarcinoma. Int J Med Sci.

[30] Ta HDK, Wang WJ, Phan NN, An Ton NT, Anuraga G, Ku SC (2021). Potential Therapeutic and Prognostic Values of LSM Family Genes in Breast Cancer. Cancers (Basel).

[31] Mering Cv, Huynen M, Jaeggi D, Schmidt S, Bork P, Snel B (2003). STRING: a database of predicted functional associations between proteins. Nucleic acids research.

[32] Shannon P, Markiel A, Ozier O, Baliga NS, Wang JT, Ramage D (2003). Cytoscape: a software environment for integrated models of biomolecular interaction networks. Genome research.

[33] Chiao CC, Liu YH, Phan NN, An Ton NT, Ta HDK, Anuraga G (2021). Prognostic and Genomic Analysis of Proteasome 20S Subunit Alpha (PSMA) Family Members in Breast Cancer. Diagnostics (Basel).

[34] Xuan DTM, Wu CC, Kao TJ, Ta HDK, Anuraga G, Andriani V (2021). Prognostic and immune infiltration signatures of proteasome 26S subunit, non-ATPase (PSMD) family genes in breast cancer patients. Aging (Albany NY).

[35] Shahi P, Wang CY, Lawson DA, Slorach EM, Lu A, Yu Y (2017). ZNF503/Zpo2 drives aggressive breast cancer progression by down-regulation of GATA3 expression. Proc Natl Acad Sci U S A.

[36] Cooke DL, McCoy DB, Halbach VV, Hetts SW, Amans MR, Dowd CF (2018). Endovascular Biopsy: In Vivo Cerebral Aneurysm Endothelial Cell Sampling and Gene Expression Analysis. Transl Stroke Res.

[37] Zhang Y, Zhang Y, Zhong C, Xiao F (2016). Cr(VI) induces premature senescence through ROS-mediated p53 pathway in L-02 hepatocytes. Scientific Reports.

[38] Zhong X, Zeng M, Bian H, Zhong C, Xiao F (2017). An evaluation of the protective role of vitamin C in reactive oxygen species-induced hepatotoxicity due to hexavalent chromium in vitro and in vivo. Journal of Occupational Medicine and Toxicology.

[39] Luczak MW, Krawic C, Zhitkovich A (2019). p53 Activation by Cr(VI): A Transcriptionally Limited Response Induced by ATR Kinase in S-Phase. Toxicol Sci.

[40] Bagchi D, Bagchi M, Stohs S (2001). Chromium (vi)-Induced Oxidative Stress, Apoptotic Cell Death and Modulation of p53 Tumor Suppressor Gene. Molecular and Cellular Biochemistry.

[41] Rager J, Ring C, Fry R, Suh M, Proctor D, Haws L (2017). High-Throughput Screening Data Interpretation in the Context of In Vivo Transcriptomic Responses to Oral Cr(VI) Exposure. Toxicological sciences: an official journal of the Society of Toxicology.

[42] Ding SZ, Yang YX, Li XL, Michelli-Rivera A, Han SY, Wang L (2013). Epithelial-mesenchymal transition during oncogenic transformation induced by hexavalent chromium involves reactive oxygen species-dependent mechanism in lung epithelial cells. Toxicol Appl Pharmacol.

[43] Zhigalenok Y, Tazhibayeva A, Kokhmetova S, Starodubtseva A, Kan T, Isbergenova D (2025). Hexavalent chromium at the crossroads of science, environment and public health. RSC Adv.

[44] Ge X, He J, Wang L, Zhao L, Wang Y, Wu G (2022). Epigenetic alterations of CXCL5 in Cr(VI)-induced carcinogenesis. Sci Total Environ.

[45] Liu Y, Deisenroth C, Zhang Y (2016). RP-MDM2-p53 Pathway: Linking Ribosomal Biogenesis and Tumor Surveillance. Trends Cancer.

[46] Kang J, Brajanovski N, Chan KT, Xuan J, Pearson RB, Sanij E (2021). Ribosomal proteins and human diseases: molecular mechanisms and targeted therapy. Signal Transduction and Targeted Therapy.

[47] Maehama T, Nishio M, Otani J, Mak TW, Suzuki A (2023). Nucleolar stress: Molecular mechanisms and related human diseases. Cancer Sci.

[48] Yang K, Yang J, Yi J (2018). Nucleolar Stress: hallmarks, sensing mechanism and diseases. Cell Stress.

[49] Wilbur S, Abadin H, Fay M, Yu D, Tencza B, Ingerman L (2012). Agency for Toxic Substances and Disease Registry (ATSDR) Toxicological Profiles. Toxicological Profile for Chromium. Atlanta (GA): Agency for Toxic Substances and Disease Registry (US).

[50] Yatera K, Morimoto Y, Ueno S, Noguchi S, Kawaguchi T, Tanaka F (2018). Cancer Risks of Hexavalent Chromium in the Respiratory Tract. Journal of UOEH.

