# Supplementary material for: Decoding Short- and Long-Term Cellular Adaptations to Cr(VI) Exposure Through High-Throughput Transcriptomics
Source: Int J Med Sci. 2026 Jan 1;23(1):113–25. doi: 10.7150/ijms.119668 (PMC12702042; doi:10.7150/ijms.119668)
Supplement: Supplementary file 1 — Supplementary figures and table. [file ijmsv23p0113s1.pdf]

Supplementary Materials

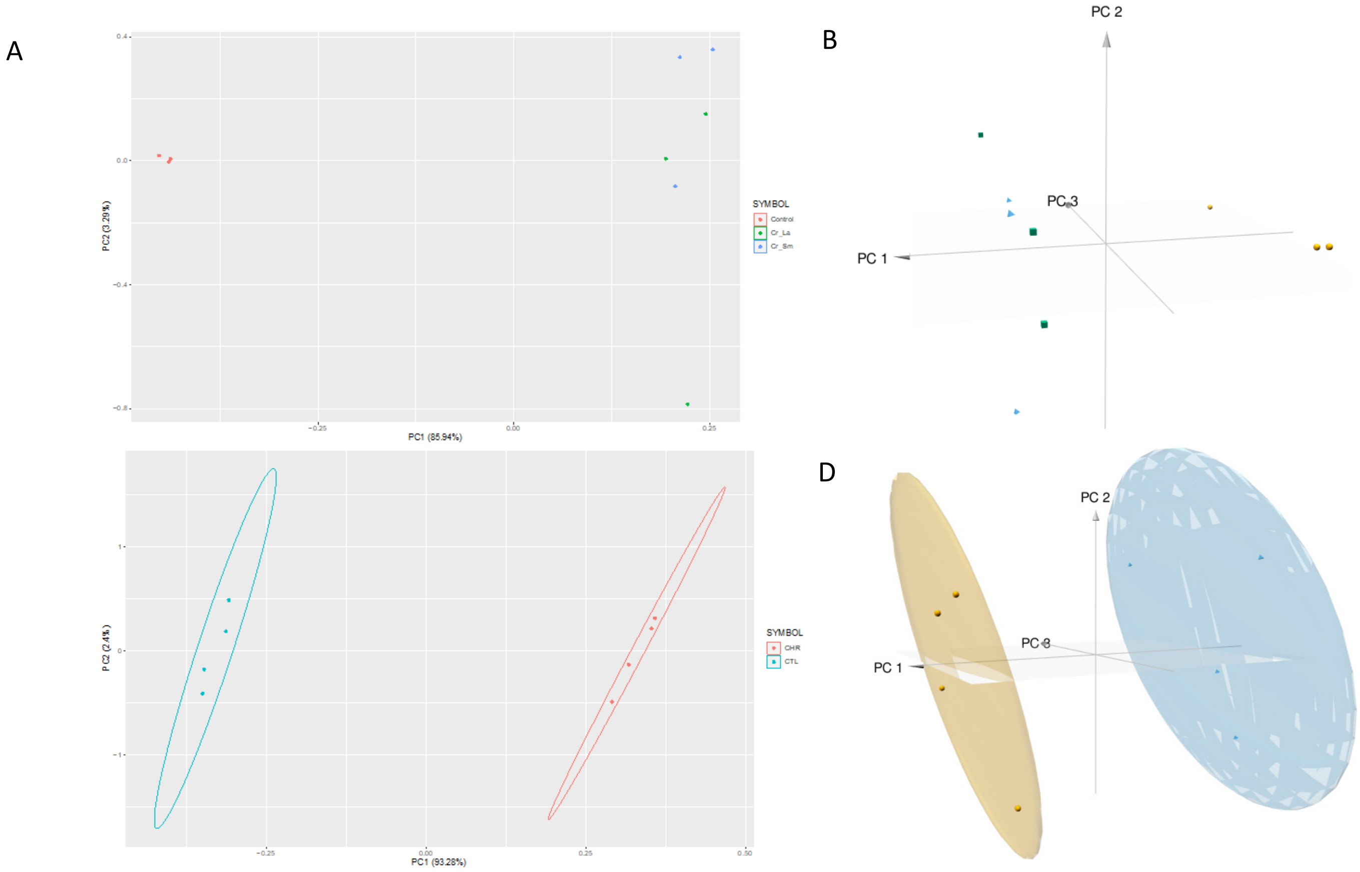

Figure S1. Principal component analysis of control and chromium treated groups.

2D and 3D PCA of control and treated groups from GSE24025 were displayed in (A-B). 2D and 3D PCA of control and treated groups from were displayed in GSE16349 (C-D). Eclipse was applied at confident interval of 0.75

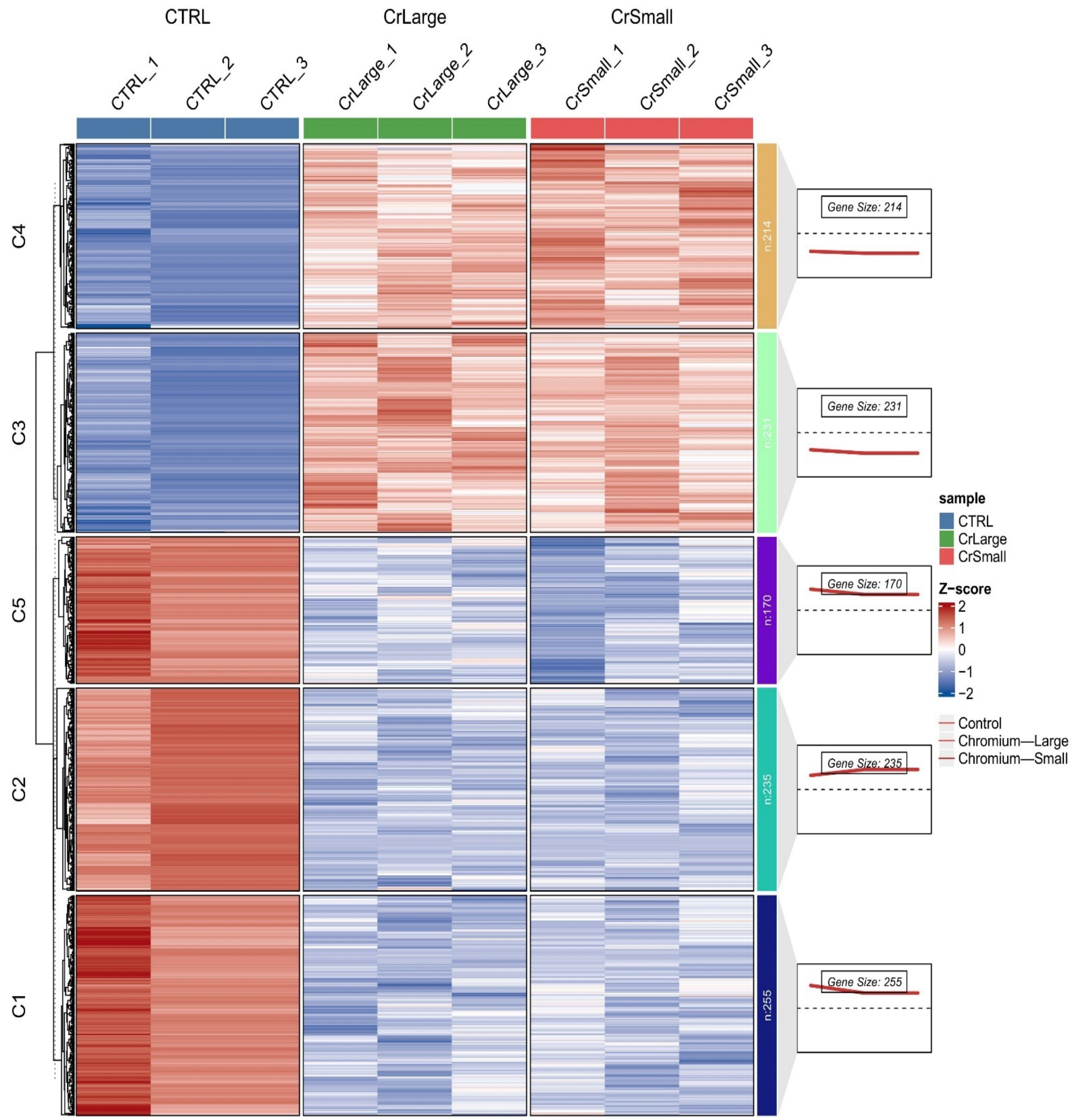

Figure S2. Hierarchical clustering corroborates Cr_large/Cr_small similarity.

Unsupervised hierarchical clustering of samples using gene-wise Z-scored expression (union of DEGs from either comparison; see Methods). Euclidean distances with Ward's linkage were used. The sample dendrogram groups Cr_large and Cr_small together within a single major clade, clearly separated from CTRL. The accompanying heatmap shows consistent expression patterns between Cr_large and Cr_small; top annotation bar indicates sample class.

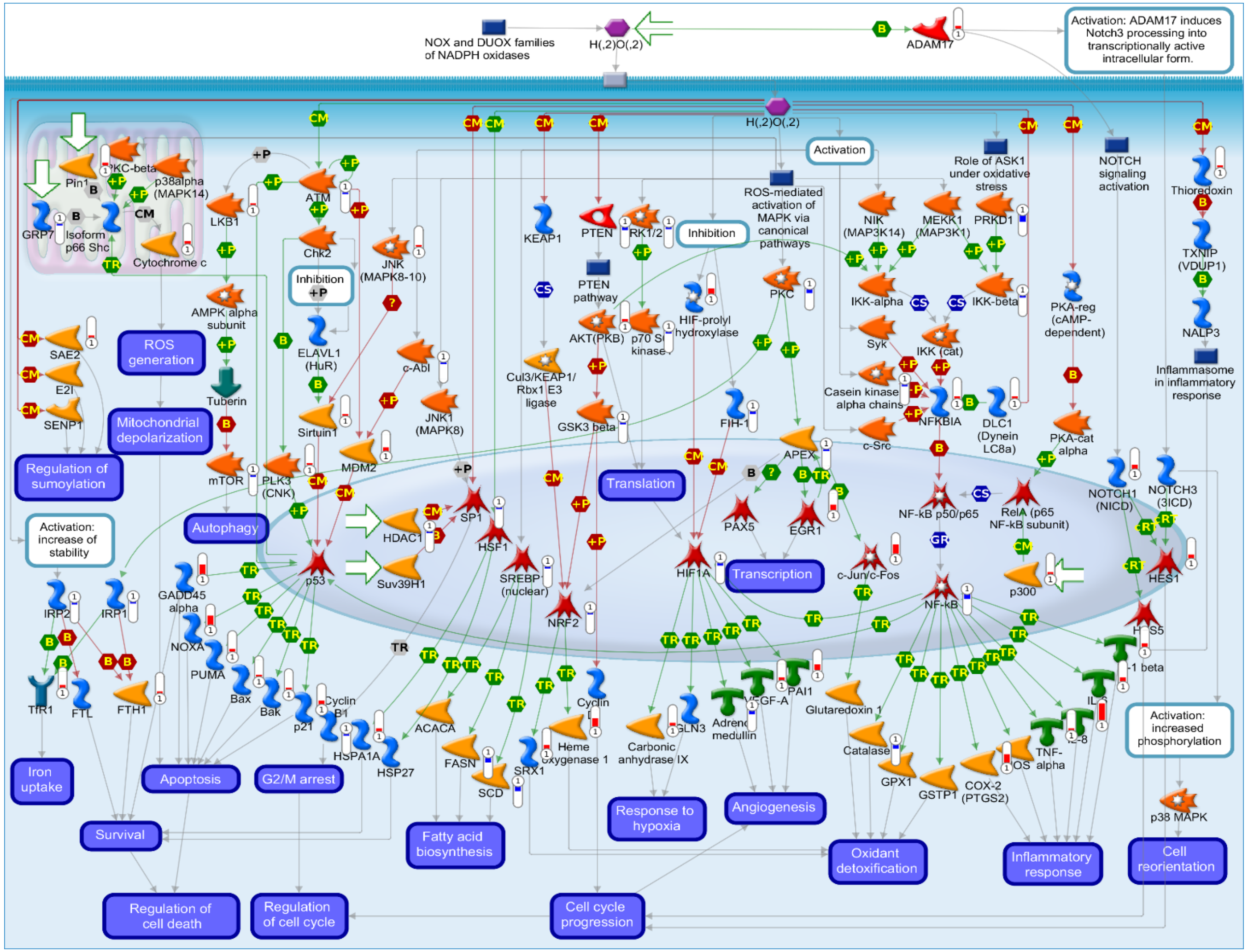

Figure S3. MetaCore pathway analysis of DEGs from GSE16349 (acute chromium-exposed vs control). (A) Top 25 enriched MetaCore Pathway Maps ranked by –log10(p-value). (B) MetaCore process map for the top-ranked pathway, “Chemotaxis_Lysophosphatidic acid signaling via GPCRs” corresponding to DEGs are overlaid and colored by direction of change (red, up-regulated; blue, down-regulated); edge styles follow MetaCore conventions (green arrows, activation; red T-bars, inhibition; dashed lines, indirect). DEGs were defined in each dataset as FC ≥ 1.2 with p-value < 0.05.

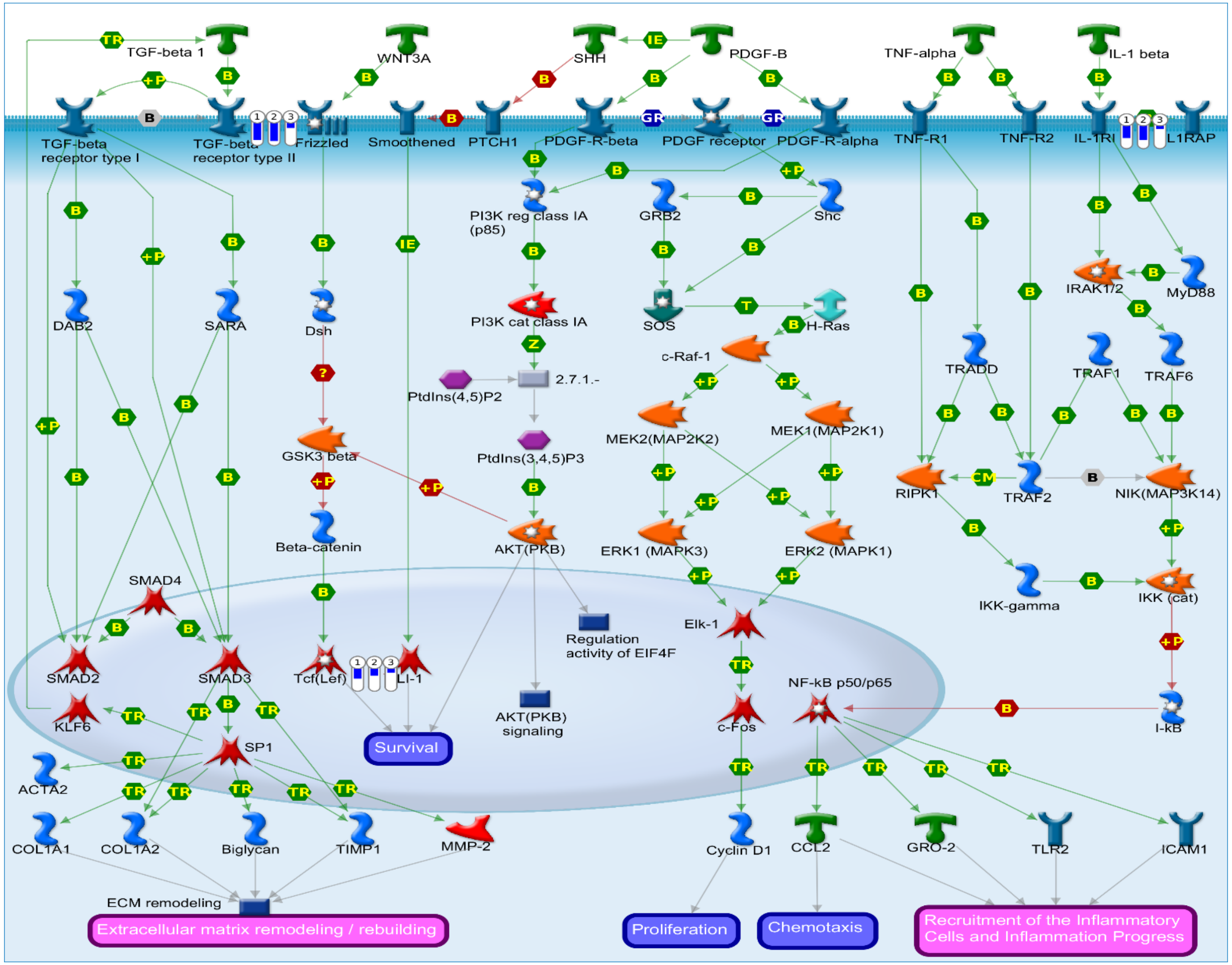

Figure S4. MetaCore pathway analysis of DEGs from GSE24025 (chronic chromium exposure vs control). (A) Top 25 enriched MetaCore Pathway Maps ranked by –log10(p-value). (B) MetaCore process map for the top-ranked pathway, "Stellate cells activation and liver fibrosis." Nodes corresponding to DEGs are overlaid and colored by direction of change (red, up-regulated; blue, down-regulated). DEGs were defined in each dataset as FC ≥ 1.2 with p-value < 0.05.

Supplementary Table 1: MetaCore pathway enrichment of shared up-regulated genes in chromium exposure. Top 50 Pathway Maps significantly enriched among genes up-regulated in both GSE16349 and GSE24025 (chromium-exposed vs. control; DEGs defined at FC ≥ 1.2 and BH-adjusted $p < 0.05$).

#	Maps	pValue	Min FDR	Network Objects from Active Data
**1**	Apoptosis and survival_Role of nuclear PI3K in NGF/ TrkA signaling	3.214E-03	1.298E-01	Ebp1, Nucleolin
**2**	Translation_Regulation of translation initiation	3.745E-03	1.298E-01	eIF1A, eIF3S7
**3**	Transport_Aldosterone-mediated regulation of ENaC sodium transport	4.611E-03	1.298E-01	ICMT, SAHH
**4**	Role of inhibition of WNT signaling in the progression of lung cancer	4.918E-03	1.298E-01	Keratin 8, CD82
**5**	Cell cycle_Role of APC in cell cycle regulation	5.235E-03	1.298E-01	ORC1L, MAD2b
**6**	G-protein signaling_H-RAS regulation pathway	6.954E-03	1.437E-01	GFRalpha1, ICMT
**7**	COVID-19: SARS-CoV-2 translation and spreading	1.544E-02	2.348E-01	NXF1, USP10
**8**	Protein folding and maturation_Insulin processing	1.704E-02	2.348E-01	SEC62, SRP-beta receptor
**9**	Mitochondrial dysfunction in neurodegenerative diseases	1.704E-02	2.348E-01	UCP2, ANT
**10**	Cell cycle_DNA replication initiation	2.165E-02	2.509E-01	ORC1L, POLA1
**11**	DNA damage_p53 activation by DNA damage	2.226E-02	2.509E-01	AATF (Che-1), USP10
**12**	dCTP/dUTP metabolism	2.606E-02	2.547E-01	POLA1, AK3
**13**	GTP-XTP metabolism	3.734E-02	2.547E-01	POLR2D, IMD1
**14**	dATP/dITP metabolism	4.041E-02	2.547E-01	POLA1, AK3
**15**	Noise-induced hair cell death and spiral ganglion neuron degeneration in deafness	4.198E-02	2.547E-01	ERM proteins, GFRalpha1
**16**	CTP/UTP metabolism	5.101E-02	2.547E-01	POLR2D, AK3
**17**	LRRK2 in neuronal apoptosis in Parkinson's disease	5.618E-02	2.547E-01	ANT
**18**	Protein folding_Membrane trafficking and signal transduction of G-alpha (i) heterotrimeric G-protein	6.258E-02	2.547E-01	ICMT
**19**	ATP/ITP metabolism	6.525E-02	2.547E-01	POLR2D, AK3
**20**	Proteolysis_Regulation of degradation of CFTR	6.577E-02	2.547E-01	Csp
**21**	Role of XBP1 protein in multiple myeloma	6.577E-02	2.547E-01	DNAJB11
**22**	Inflammatory response in ischemia-reperfusion injury during myocardial infarction	6.894E-02	2.547E-01	HSP60
**23**	Transport_CFTR sorting endosome	7.211E-02	2.547E-01	USP10
**24**	CFTR folding and maturation (normal and cystic fibrosis)	7.841E-02	2.547E-01	Csp

**25**	Putative pathways of activation of monoclonal protein secretion in multiple myeloma	8.154E-02	2.547E-01	DNAJB11
**26**	G-protein signaling_K-RAS regulation pathway	8.154E-02	2.547E-01	ICMT
**27**	CREB1-dependent transcription deregulation in Huntington's Disease	8.466E-02	2.547E-01	UCP2
**28**	Cell cycle_Initiation of mitosis	8.466E-02	2.547E-01	Nucleolin
**29**	Neurophysiological process_GABA-A receptor life cycle	8.777E-02	2.547E-01	Ubiquilin-1
**30**	CFTR sorting endosome in cystic fibrosis	8.777E-02	2.547E-01	USP10
**31**	Putative role of Tregs in COPD	9.705E-02	2.547E-01	IL7RA
**32**	Role of IL-6 in obesity and type 2 diabetes in adipocytes	1.032E-01	2.547E-01	UCP2
**33**	DNA damage_Mismatch repair	1.032E-01	2.547E-01	USP10
**34**	G-protein signaling_N-RAS regulation pathway	1.062E-01	2.547E-01	ICMT
**35**	Role of GSK3 beta in cardioprotection against myocardial infarction	1.062E-01	2.547E-01	ANT
**36**	Development_Role of cell-cell and ECM-cell interactions in oligodendrocyte differentiation and myelination	1.093E-01	2.547E-01	CD82
**37**	G-protein signaling_G-Protein alpha-q signaling	1.093E-01	2.547E-01	RGS2
**38**	Immune response_TLR ligands	1.123E-01	2.547E-01	HSP60
**39**	NALP3 inflammasome activation in age-related macular degeneration (AMD)	1.123E-01	2.547E-01	VDAC 2
**40**	Glucose-excited neurons of arcuate nucleus in obesity (rodent model)	1.123E-01	2.547E-01	UCP2
**41**	Urea cycle	1.153E-01	2.547E-01	SLC25A15 (ORC1)
**42**	Upregulation of MITF in melanoma	1.153E-01	2.547E-01	TBX2
**43**	G-protein signaling_G-Protein alpha-s signaling	1.153E-01	2.547E-01	RGS2
**44**	Cell cycle_The metaphase checkpoint	1.153E-01	2.547E-01	MAD2b
**45**	Immune response_Generation of memory CD4+ T cells	1.183E-01	2.547E-01	IL7RA
**46**	Transcription_Assembly of RNA Polymerase II preinitiation complex	1.213E-01	2.547E-01	POLR2D
**47**	Development_Oligodendrocyte differentiation (general schema)	1.213E-01	2.547E-01	CD82
**48**	Regulation of degradation of CFTR in cystic fibrosis	1.243E-01	2.547E-01	Csp
**49**	De novo IMP biosynthesis	1.243E-01	2.547E-01	PRPS2
**50**	Role of type 2 innate lymphoid cells in airway allergic inflammation and tissue repair	1.243E-01	2.547E-01	IL7RA

Supplementary Table 2: MetaCore pathway enrichment of shared down-regulated genes in chromium exposure. Top 50 Pathway Maps significantly enriched among genes up-regulated in both GSE16349 and GSE24025 (chromium-exposed vs. control; DEGs defined at FC ≤ −1.2 and BH-adjusted $p < 0.05$).

#	Maps	pValue	Min FDR	Network Objects from Active Data
**1**	Regulation of metabolism_Glucocorticoid receptor signaling in glucose and lipid metabolism	2.298E-04	4.958E-02	SETDB2, GCR Alpha, GCR, LIPIN1, NF-I
**2**	DNA damage_ATM/ATR regulation of G2/M checkpoint: nuclear signaling	2.382E-04	4.958E-02	Cyclin B2, p53BP1, Cyclin A, Cyclin B
**3**	Cytoskeleton remodeling_Regulation of actin cytoskeleton nucleation and polymerization by Rho GTPases	2.833E-04	4.958E-02	RhoB, DRF, RhoA-related, DAAM1
**4**	Cell cycle_Cell cycle (generic schema)	3.926E-04	5.153E-02	Cyclin A, Cyclin B, p130
**5**	Inhibition of oligodendrocyte precursor cells differentiation by Wnt signaling in multiple sclerosis	6.654E-04	6.049E-02	TCF7L2 (TCF4), GLI-3R, GLI-3
**6**	G-protein signaling_RhoB activation	6.913E-04	6.049E-02	PIAS1, RhoB, GCR, TGF-beta receptor type II
**7**	Cell cycle_Regulation of G1/S transition (part 2)	9.336E-04	7.002E-02	Cyclin A2, Cyclin A, p130
**8**	SHH signaling in colorectal cancer	1.262E-03	8.082E-02	TCF7L2 (TCF4), GLI-3R, GLI-3
**9**	Upregulation of IL-8 expression in colorectal cancer	1.385E-03	8.082E-02	TCF7L2 (TCF4), IL-1RI, TGF-beta receptor type II
**10**	Signal transduction_FGFR4 signaling	2.512E-03	1.266E-01	HMDH, SC4MOL, Rab-11A, INSIG1
**11**	WNT signaling in hepatocellular carcinoma (HCC)	2.652E-03	1.266E-01	TCF7L2 (TCF4), Tcf(Lef), Prickle-1
**12**	Signal transduction_WNT/Beta-catenin signaling in tissue homeostasis	3.050E-03	1.335E-01	TCF7L2 (TCF4), Tcf(Lef), Cyclin A2
**13**	SCAP/SREBP Transcriptional Control of Cholesterol and FA Biosynthesis	3.714E-03	1.484E-01	HMDH, ELOVL6, INSIG1
**14**	Development_VEGF signaling via VEGFR2 - generic cascades	3.958E-03	1.484E-01	TCF7L2 (TCF4), p90Rsk, SHB, p120GAP
**15**	Glucocorticoids-mediated inhibition of pro-constrictory and pro-inflammatory signaling in asthmatic airway smooth muscle cells	4.728E-03	1.655E-01	GCR Alpha, GCR, GCR Beta
**16**	Signal transduction_Angiotensin II/ AGTR1 signaling via p38, ERK and PI3K	5.124E-03	1.681E-01	MAP2K5 (MEK5), PDGF-C, ATP7A, p90Rsk
**17**	Immune response_Lysophosphatidic acid signaling via NF-kB	5.587E-03	1.719E-01	PLC-epsilon, p90Rsk, CARD10
**18**	DNA damage_ATR activation by DNA damage	5.893E-03	1.719E-01	p53BP1, NEK1, p90Rsk

**19**	Glucocorticoid-induced elevation of intraocular pressure as glaucoma risk factor	9.097E-03	2.190E-01	GCR Alpha, GCR, GCR Beta
**20**	Transcription_Sirtuin6 regulation and functions	9.097E-03	2.190E-01	HMDH, ELOVL6, AMPK gamma subunit
**21**	Development_Beta-catenin destruction complex signaling	9.177E-03	2.190E-01	TCF7L2 (TCF4), Tcf(Lef)
**22**	Cell cycle_Chromosome condensation in prometaphase	9.177E-03	2.190E-01	Cyclin A, Cyclin B
**23**	Development_Non-canonical TGF-beta signaling via PI3K, RhoA, and ROS	1.035E-02	2.363E-01	RhoB, RhoA-related, TGF-beta receptor type II
**24**	Action of GSK3 beta in bipolar disorder	1.096E-02	2.397E-01	TCF7L2 (TCF4), Tcf(Lef)
**25**	Signal transduction_AKT(PKB) activation	1.170E-02	2.403E-01	PIAS1, IL-1RI, Cyclin A
**26**	G-protein signaling_CDC42 inhibition	1.190E-02	2.403E-01	ARHGAP10, ARHGAP21
**27**	Stellate cells activation and liver fibrosis	1.266E-02	2.461E-01	IL-1RI, Tcf(Lef), TGF-beta receptor type II
**28**	CAR signaling via cross-talk / Human Version	1.389E-02	2.462E-01	GCR, INSIG1
**29**	CAR signaling via cross-talk / Rodent version	1.493E-02	2.462E-01	GCR, INSIG1
**30**	Inhibition of TGF-beta 1 signaling in early colorectal cancer	1.493E-02	2.462E-01	SMAD6, TGF-beta receptor type II
**31**	Impaired inhibition of Th17 cell differentiation by IFN-beta in multiple sclerosis	1.601E-02	2.462E-01	IL-1RI, TGF-beta receptor type II
**32**	Glucocorticoid- and LABA-mediated inhibition of pro-inflammatory signaling in asthmatic airway fibroblasts/myofibroblasts	1.601E-02	2.462E-01	IL-1RI, GCR
**33**	Suppression of TGF-beta signaling in pancreatic cancer	1.712E-02	2.462E-01	SMAD6, TGF-beta receptor type II
**34**	Immune response_M-CSF-receptor signaling	1.749E-02	2.462E-01	MAP2K5 (MEK5), Tcf(Lef), p120GAP
**35**	Transport_Aldosterone-mediated regulation of ENaC sodium transport	1.826E-02	2.462E-01	GCR, DSIPI (GILZ)
**36**	Role of histone modifiers in progression of multiple myeloma	1.826E-02	2.462E-01	UTX, HDAC8
**37**	Deregulation of canonical WNT signaling in major depressive disorder	1.943E-02	2.462E-01	TCF7L2 (TCF4), Tcf(Lef)
**38**	Development_WNT/Beta-catenin signaling in organogenesis	1.943E-02	2.462E-01	TCF7L2 (TCF4), Tcf(Lef)
**39**	Role of inhibition of WNT signaling in the progression of lung cancer	1.943E-02	2.462E-01	MAP2K5 (MEK5), DAAM1
**40**	Inhibition of TGF-beta signaling in lung cancer	1.943E-02	2.462E-01	SMAD6, TGF-beta receptor type II
**41**	DNA damage_Double-strand break repair via homologous recombination	2.056E-02	2.462E-01	p53BP1, NEK1, SMARCAD1

**42**	Obesity: putative pathways for stimulation of fat cell differentiation by Bisphenol A	2.063E-02	2.462E-01	TCF7L2 (TCF4), GCR
**43**	Stem cells_Cooperation between Hedgehog, IGF-2 and HGF signaling pathways in medulloblastoma stem cells	2.063E-02	2.462E-01	TCF7L2 (TCF4), Tcf(Lef)
**44**	Cell cycle_Role of APC in cell cycle regulation	2.063E-02	2.462E-01	Cyclin A, Cyclin B
**45**	Immune response_IFN-alpha/beta signaling via MAPKs	2.186E-02	2.491E-01	PIAS1, TCF7L2 (TCF4), p130
**46**	Development_HGF-dependent inhibition of TGF-beta-induced EMT	2.313E-02	2.491E-01	p90Rsk, TGF-beta receptor type II
**47**	Development_Negative regulation of WNT/Beta-catenin signaling in the nucleus	2.391E-02	2.491E-01	TCF7L2 (TCF4), GLI-3R, Tcf(Lef)
**48**	Immune response_Th17 cell differentiation	2.442E-02	2.491E-01	IL-1RI, TGF-beta receptor type II
**49**	Mechanisms of cell adhesion-mediated-drug resistance (CAM-DR) in multiple myeloma	2.442E-02	2.491E-01	HMDH, Cyclin A
**50**	Higher ESR1 / ESR2 ratio in breast cancer	2.442E-02	2.491E-01	Cyclin A2, p130

Supplementary Table 3: MetaCore pathway enrichment of specific DEGs in acute chromium exposure. Top 50 Pathway Maps significantly enriched among DEGs in GSE16349 (chromium-exposed vs. control; DEGs defined at $|FC| \geq 1.2$ and BH-adjusted $p < 0.05$).

#	Maps	pValue	Min FDR	Network Objects from Active Data
**1**	Chemotaxis_Lysophosphatidic acid signaling via GPCRs	6.811E-21	1.035E-17	cPKC (conventional), G-protein alpha-12 family, AKT1, PLD1, c-Fos, LPAR1, PI3K cat class IA (p110-beta), Tcf(Lef), IL-8, EGFR, G-protein alpha-i family, H-Ras, F-Actin cytoskeleton, c-Jun, MKL2, ERK1/2, PRK1, HB-EGF, Beta-catenin, EGR1, CRK, Caspase-9, G-protein alpha-q/11, p21, PKC-epsilon, LIMK, TRIP6, Paxillin, Rac1, IP3 receptor, ATF-2/c-Jun, G-protein gamma 12, LPAR2, ROCK, PRKD1, PI3K reg class IA (p85), Bax, AP-1, GSK3 beta, PAK, p70 S6 kinase1, PKC, PLC-beta, FKHR, Vinculin, ATF-2, HAS2, LPAR6, Caspase-3, FasR(CD95), Cyr61, MDM2, FAK1, SIVA1, CREB1, Bcl-XL, MEK1/2, G-protein beta/gamma, Rho GTPase, CDC42, Actin cytoskeletal, N-CoR, IL13RA2, JNK(MAPK8-10), MKL1, CTGF, AKT(PKB), PDK (PDPK1), ADAM17, PLC-epsilon, Elk-1, SRF, Cofilin, mTOR, PREX1
**2**	Oxidative stress_ROS signaling	1.236E-19	9.389E-17	Casein kinase II, alpha chains, NOTCH1 (NICD), Thioredoxin, Heme oxygenase 1, PLK3 (CNK), SREBP1 (nuclear), Adrenomedullin, IRP1, SCD, HIF1A, IL-8, SRX1, ERK1/2, EGR1, c-Jun/c-Fos, VEGF-A, Pin1, p21, Bak, Cytochrome c, p300, FASN, Carbonic anhydrase IX,

				NFKBIA, HSPA1A, TfR1, COX-2 (PTGS2), IL-1 beta, PRKD1, Bax, Sirtuin1, GSK3 beta, p70 S6 kinase1, PKC, LKB1, PTEN, HES1, FTH1, ATM, IKK-beta, DLC1 (Dynein LC8a), IRP2, GRP75, MDM2, c-Abl, IL-6, GADD45 alpha, NOXA, JNK(MAPK8-10), HIF-prolyl hydroxylase, HDAC1, NF-kB, FIH-1, SAE2, AKT(PKB), SP1, Catalase, Cyclin B1, NRF2, PUMA, ADAM17, APEX, PAI1, mTOR, TNF-alpha
**3**	Transcription_HIF-1 targets	4.375E-18	2.215E-15	G3P2, PDK1, ARNT, NIX, Heme oxygenase 1, Lysyl oxidase, ROR-alpha, Adrenomedullin, CITED2, PLGF, HIF1A, P4HA2, HIF-1, PFKL, REDD1, SLC9A1, GPI, Endoglin, VEGF-A, P4HA1, Cyclin G2, GLUT3, ENO1, p21, Thrombospondin 1, MSH6, Carbonic anhydrase IX, PGK1, Stanniocalcin 2, NIP3, AK3, IBP3, TfR1, ID2, Mxi1, Adipophilin, GLUT1, Carbonic anhydrase XII, ALDOA, F263, HXK2, LOXL4, MMP-2, TGM2, NOXA, PLAUR (uPAR), CTGF, MCT4, LDHA, DEC1 (Stra13), ALDOC, c-Myc, Mcl-1, PAI1, SDF-1, CXCR4, LRP1, PKM2
**4**	Cytoskeleton remodeling_Regulation of actin cytoskeleton organization by the kinase effectors of Rho GTPases	1.474E-15	4.516E-13	BETA-PIX, WRCH-1, RhoJ, Destrin, F-Actin cytoskeleton, Spectrin, SLC9A1, PRK1, Caldesmon, Alpha-actinin, CPI-17, Alpha adducin, RhoC, RhoB, MyHC, LIMK, Rac3, Paxillin, Rac1, MRCK, RhoGDI alpha, Talin, GIT1, ROCK, MRCKalpha, PAK, Cofilin, non-muscle, Rac1-related, Cdc42 subfamily, Vinculin, MSN (moesin), ERM proteins, MRLC, RhoA-related, Cortactin, CDC42, Actin cytoskeletal, Filamin A, TC10, Cofilin
**5**	Signal transduction_PDGF signaling via PI3K/AKT and NFkB pathways	1.486E-15	4.516E-13	PDK1, GRB2, c-Fos, K-RAS, SOD2, HIF1A, PI3K cat class IA (p110-beta), ETS1, c-Jun, ERK1/2, ETS2, Beta-catenin, YB-1, c-Jun/Fra-1, PDGF-R-alpha, DDX5, NFKBIA, Rac1, PI3K reg class IA (p85), p120GAP, GSK3 beta, Calmodulin, PTEN, FKHR, HXK2, Tenascin-C, MMP-2, c-Abl, PDGF-C, Transgelin, PDGFR-ab, NF-kB, MKL1, AKT(PKB), SP1, PDGF receptor, PDK (PDPK1), p27KIP1, c-Myc, Elk-1, SRF, PDGF-D, Phox1 (PRRX1), mTOR, PDGF-R-beta
**6**	Cell cycle_DNA replication initiation	6.945E-15	1.758E-12	MCM3, WDR18, Cyclin B, ORC2L, CDC7, ORC1L, Histone H3, GINS2, MCM5, RPA3, MCM4, E2F1, GINS complex, ORC3L, GCN5, Jade-1, Importin (karyopherin)-alpha, SSRP1, ORC6L, POLA1, Geminin, CDK1 (p34), CDK2, NOC3L, MCM7, SIK, SLD5, Treslin, PP1-cat alpha, RecQL4, TRF2, SMARCA5, RPA1, ORC5L, MCM2, GRWD1, Cdt1, HP1 alpha, PLK1, RPA2, Histone H4, RFWD3, CDC45L

**7**	Apoptosis and survival_p53 and p73-dependent apoptosis	2.199E-14	4.772E-12	Cathepsin L, NIX, DR5(TNFRSF10B), p14ARF, Sin3A, Caspase-6, PLEKHF1, SOD2, iASPP, HIPK2, ARIH2 (TRIAD1), Apaf-1, ASPP1, E2F1, DAXX, Prohibitin, Pin1, IL4RA, Bak, p300, NIP3, BAD, ZNHIT1, Bax, Sirtuin1, Survivin, PTEN, DIP, PCAF, USP7, Caspase-3, FasR(CD95), MDM2, c-Abl, SIVA1, Bcl-XL, GADD45 alpha, NOXA, NOL3, Mitofusin 2, AKT(PKB), DR4(TNFRSF10A), DNM1L (DRP1), Caspase-1, Mcl-1, PUMA, APEX, XAF1, Bid
**8**	Transcription_Negative regulation of HIF1A function	2.074E-13	3.938E-11	PLK3 (CNK), MCM3, FHL3, p14ARF, Casein kinase I delta, MCM5, RUNX3, CITED2, HIF1A, FBXW7, SART1, KLF2, ARD1, VCP, PSMA7, SKP1, HSP90 beta, Cul1/Rbx1 E3 ligase, AML1 (RUNX1), Ubiquitin, Sirtuin1, GSK3 beta, EGLN2, MCM7, FHL1 (SLIM1), FHL2, PTEN, Sirtuin7, SSAT, LAMP2, Elongin C, OS-9, MDM2, HIF-prolyl hydroxylase, FIH-1, MCM2, HSP90, Calpain 1(mu), PRDX4, HSP70, RUVBL2, EGLN1
**9**	Signal transduction_PDGF signaling via MAPK cascades	4.654E-13	7.855E-11	SPHK1, Osteopontin, MMP-13, GRB2, c-Fos, Fra-1, H-Ras, c-Jun, ERK1/2, EGR1, PDGF-R-alpha, p21, p90Rsk, Rac1, COX-2 (PTGS2), GIT1, AP-1, MAPKAPK2, JunD, VAV-2, MMP-2, FAK1, c-Abl, PDGF-C, CREB1, IL-6, MEK1/2, JNK(MAPK8-10), PDGFR-ab, ERK2 (MAPK1), SP1, PDGF receptor, Stromelysin-1, p27KIP1, c-Myc, Elk-1, GRB10, PDGF-D, Phox1 (PRRX1), PDGF-R-beta
**10**	Development_Positive regulation of WNT/Beta-catenin signaling in the nucleus	1.250E-12	1.898E-10	Casein kinase II, alpha chains, SMYD2, SOX11, TBL1X, CBP, Tcf(Lef), beta-TrCP, TCF7L2 (TCF4), FOXM1, USP5, Jade-1, Beta-catenin, BCL9/B9L, VCP, Pin1, TLE, ZIP-kinase, p300, CBP/P300, Dsh, TDG, Cul1/Rbx1 E3 ligase, NLK, Kindlin-2, Sirtuin1, GSK3 beta, CARF, FHL2, WIP1, PCAF, RUNX, WNT, TBLR1, SOX4, HDAC1, APPL, ERK2 (MAPK1), RUVBL2, LRRFIP2, SOX9, Frizzled
**11**	Signal transduction_mTORC1 downstream signaling	3.205E-12	4.425E-10	SC5D, PDK1, Rictor, SREBP1 (nuclear), LIPIN1, G6PD, SCD, HIF1A, IRS-1, MAF1, Cyclin D, eEF2K, VEGF-A, HMGCS2, CBP80, Cytochrome c, SREBP2 (nuclear), MVK, ULK2, UBF, BAD, RRN3, p70 S6 kinases, GLUT1, GSK3 beta, p70 S6 kinase1, MTHFD2, ACSL3, ATG13, MDM2, p70 S6 kinase2, p27KIP1, YY1, PPAR-gamma, PFKP, GRB10, PDIP46
**12**	DNA damage_ATM activation by DNA damage	4.621E-12	5.849E-10	Casein kinase II, alpha chains, RCAD, CDK5R1 (p35), p14ARF, PARP-1, BRAT1, Histone H3, OBFC2B, EGFR, RAD17, INTS3, Histone H2B, Aven, CDK5R1 (p25), PP2A regulatory, Rad50, PP2A catalytic, PPP2R3A, HSP90 beta,

				p18, Pellino 1, DMAP1, IHPK2, SOSSC, NK31, MYST1, ATM, Itch, Casein kinase II, alpha chain (CSNK2A1), MDM2, c-Abl, RecQL4, TTI2, eIF3S6, HDAC1, SKP2, HSP90, Calpain 1(mu), Histone H1.2, Histone H4, NDR1 (STK38)
**13**	Development_EGFR signaling	8.548E-12	9.988E-10	NOTCH1 (NICD), ITGB1, JAK2, PI3K cat class IA, NOTCH1 receptor, NCK1, GRB2, c-Fos, MMP-1, EGFR, H-Ras, c-Jun, ERK1/2, HB-EGF, EGR1, CRK, p90Rsk, NFKBIA, Paxillin, STAT1, CDK2, BAD, EGR2 (Krox20), PI3K reg class IA (p85), AP-1, GSK3 beta, p70 S6 kinase1, PI3K reg class IA, CDK4, HES1, WNT, Caspase-3, RIPK1, MMP-2, FAK1, MEK1/2, NF-kB, CTGF, ERK2 (MAPK1), AKT(PKB), NOTCH1 (NEXT), c-Myc, Mcl-1, Elk-1, mTOR
**14**	Signal transduction_S1P1 receptor signaling	1.276E-11	1.315E-09	SPHK1, MEK1(MAP2K1), S1P1 receptor, AKT1, PI3K reg class IA (p85-alpha), GRB2, c-Fos, PI3K cat class IA (p110-beta), KLF5, Tcf(Lef), EGFR, G-protein alpha-i family, ZO-1, H-Ras, c-Jun, ERK1/2, Beta-catenin, c-Jun/c-Fos, VEGF-A, Alpha-actinin, CRK, G-protein alpha-i3, PKC-epsilon, G-protein alpha-i2, Paxillin, Rac1, IP3 receptor, COX-2 (PTGS2), IL-1 beta, G-protein alpha-i1, p70 S6 kinases, AP-1, GSK3 beta, MAPKAPK2, Calmodulin, PLC-beta, PKC-alpha, Adenylate cyclase, Cyr61, MMP-14, FAK1, IL-6, Cortactin, MEK1/2, G-protein beta/gamma, CDC42, Fyn, JNK(MAPK8-10), CTGF, Osteoprotegerin, AKT(PKB), Beta-arrestin1, PDK (PDPK1), Cofilin, mTOR, PDGF-R-beta, TNF-alpha
**15**	Signal transduction_ESR1 (membrane) and ESR2 (membrane) signaling	1.299E-11	1.315E-09	CBP, cPKC (conventional), PI3K cat class IA, PI3K reg class IA (p85-alpha), GRB2, ROCK2, IRS-1, Tcf(Lef), C/EBPbeta, EGFR, G-protein alpha-i family, H-Ras, ERK1/2, HB-EGF, Beta-catenin, EGR1, Guanylate cyclase A (NPR1), G-protein alpha-i3, FASN, G-protein alpha-i2, p90Rsk, Rac1, IP3 receptor, G-protein alpha-13, SREBP1 precursor, G-protein alpha-i1, PI3K reg class IA (p85), DMRT1, p120GAP, GSK3 beta, Profilin, Calmodulin, PKC, LKB1, PI3K reg class IA, PLC-beta, PKC-alpha, MSN (moesin), CTH, Adenylate cyclase, MMP-2, FAK1, CREB1, MEK1/2, G-protein beta/gamma, CDC42, EZH2, G-protein alpha-q, Striatin, AKT(PKB), PDK (PDPK1), p27KIP1, Elk-1, SRF, FNBP1L, Cofilin
**16**	Development_Cytokine-mediated regulation of megakaryopoiesis	1.478E-11	1.403E-09	GAB2, JAK2, PI3K cat class IA, IL-1 alpha, Cyclin D3, GRB2, ITGA2B, PP1-cat, ERK1/2, p21, IRF1, STAT1, IL-1 beta, PI3K reg class IA (p85), GP-IB alpha, GSK3 beta, p70

				S6 kinase1, PI3K reg class IA, MafB, alpha-IIb/beta-3 integrin, LIF, c-MPL, IL-1RI, IL-6, Bcl-XL, STAT5, NF-kB, AKT(PKB), SP1, p27KIP1, c-Myc, SDF-1, CXCR4, IL-11, mTOR
**17**	DNA damage_p53 activation by DNA damage	2.055E-11	1.836E-09	PLK3 (CNK), CBP, USP10, SMG1, SIAH2, HIPK2, E2F1, DAXX, 14-3-3, PP2A regulatory, AATF (Che-1), MARKK, PP2A catalytic, p21, p300, P53DINP1a, Chk1, Bax, Sirtuin1, DDB2, Cullin 4A, TTC5 (Strap), PCAF, PP2A structural, USP7, MYST1, ATM, MDM2, c-Abl, Bcl-XL, COP1, GADD45 alpha, NOXA, JNK(MAPK8-10), FBXO31, PUMA, XAF1, PP2C gamma, RFWD3
**18**	TGF-beta 1-induced transactivation of membrane receptors signaling in hepatocellular carcinoma (HCC)	2.255E-11	1.853E-09	alpha-3/beta-1 integrin, ITGB1, PI3K cat class IA, TGF-beta receptor type II, DOCK1, Beta-catenin, alpha-5/beta-1 integrin, CRK, PDGF-R-alpha, Fibronectin, DDX5, Actin, Rac1, CDK2, GSK3 beta, ITGA2, PI3K reg class IA, CDK4, PTEN, SLUG, FAK1, c-Abl, alpha-2/beta-1 integrin, Cyclin A, SNAIL1, AKT(PKB), PDGF receptor, ITGA5, c-Myc, Axin, Cofilin, PDGF-R-beta
**19**	Signal transduction_Non-canonical WNT5A signaling	2.317E-11	1.853E-09	MEK1(MAP2K1), cPKC (conventional), Casein kinase I delta, ROR2, AKT1, Calcineurin A (catalytic), DAAM1, MMP-1, IL-8, G-protein alpha-i family, TCF7L2 (TCF4), c-Jun, ERK1/2, Beta-catenin, FZD4, RYK, Paxillin, Rac1, IP3 receptor, NLK, G-protein alpha-i1, ROCK, WNT5A, FZD2, Calmodulin, PLC-beta, JunD, PKC-lambda/iota, ATF-2, G-protein alpha-t2, G-protein beta/gamma, CDC42, G-protein alpha-q, JNK(MAPK8-10), JNK2(MAPK9), Filamin A, LIMK2, SP1, PDE6 cat, FZD7, DVL-2, Elk-1, TAK1(MAP3K7), Cofilin
**20**	Development_NOTCH signaling inhibition	3.122E-11	2.351E-09	Casein kinase II, alpha chains, NOTCH1 (NICD), BLOS2, AKT1, NOTCH1 receptor, BACE1, FBXW7, RITA1, Presenilin 2, DYRK1a, HDAC4, LSD1, EGFL9, NOTCH2 receptor, L3MBTL3, EGFL7, Cul1/Rbx1 E3 ligase, CHAC1, NOTCH2 (2ICD), NLK, ETO, Sirtuin1, GSK3 beta, FHL1 (SLIM1), Cyclin C, RUNX2, NEURL1, TACC3, RING1, Itch, Elongin C, MMP-14, NOTCH1 precursor, MDM2, N-CoR, Skp2/TrCP/FBXW, SNAIL1, USP12, HDAC1, AKT(PKB), ARRDC1, Beta-arrestin1, DEC1 (Stra13), PLK1, DVL-2, SHARP (SPEN), APP-C59 (AICD), NUMB, CBX4
**21**	Signal transduction_IGF-1 receptor signaling	3.250E-11	2.351E-09	JAK2, 14-3-3 epsilon, PI3K cat class IA, GRP78, Calcineurin A (catalytic), SREBP1 (nuclear), RHEB2, GRB2, IRS-2, SOD2, SCD, IRS-1, ASK1 (MAP3K5), G-protein alpha-i

				family, H-Ras, ERK1/2, 14-3-3 gamma, Beta-catenin, EGR1, p300, FASN, PKC-epsilon, p90Rsk, PLC-beta1, NIP3, BAD, 14-3-3 beta/alpha, I-kB, G-protein alpha-i1, PI3K reg class IA (p85), GSK3 beta, p70 S6 kinase1, RUNX2, FKHR, PCAF, HMDH, IGF-2, MMP-14, MMP-2, FAK1, Bcl-XL, MNK2(GPRK7), MEK1/2, G-protein beta/gamma, AKT(PKB), Catalase, Beta-arrestin1, PDK (PDPK1), NRF2, p27KIP1, LRP1, mTOR
**22**	Immune response_B cell antigen receptor (BCR) pathway	5.023E-11	3.468E-09	MEK1(MAP2K1), LRRK1, Calcineurin A (catalytic), NCK1, GRB2, c-Fos, NF-kB1 (p50), K-RAS, ETS1, H-Ras, PIP5KIII, ERK1/2, CIN85, EGR1, CD79 complex, PP2A catalytic, Fibronectin, NFKBIA, PI3K cat class IA (p110-delta), Rac1, IP3 receptor, CDK6, BAD, PI3K reg class IA (p85), GSK3 beta, p70 S6 kinase1, Calmodulin, CD79A, CalDAG-GEFIII, CDK4, FKHR, ATF-2, MALT1, N-Ras, IKK-beta, CD79B, VAV-2, MEK2(MAP2K2), GSK3 alpha/beta, IKK-gamma, Bcl-XL, MEK1/2, CDC42, WASP, Actin cytoskeletal, Bcl-10, NF-kB, AKT(PKB), PDK (PDPK1), Elk-1, TAK1(MAP3K7), mTOR, MEKK4(MAP3K4)
**23**	Leptin signaling in colorectal cancer	6.791E-11	4.485E-09	JAK2, PI3K cat class IA, c-Fos, IL-8, TCF7L2 (TCF4), c-Jun, ERK1/2, Beta-catenin, VEGF-A, ABIN-2, NFKBIA, Rac1, CDK1 (p34), GSK3 beta, p70 S6 kinase1, Survivin, PI3K reg class IA, PTEN, FAK1, IL-6, Bcl-XL, MEK1/2, CDC42, JNK(MAPK8-10), AKT(PKB), Cyclin B1, c-Myc, ADAM17, mTOR
**24**	Neurogenesis_NGF/ TrkA MAPK-mediated signaling	7.986E-11	4.876E-09	SPHK1, GAB2, CDK5R1 (p35), ERK5 (MAPK7), RIN, GRB2, c-Fos, NF-kB1 (p50), K-RAS, Fra-1, SAC, H-Ras, c-Jun, ERK1/2, HB-EGF, CrkL, EGR1, PP2A regulatory, CRK, PP2A catalytic, DNAJA3 (TID1), p21, VGF, C3G, PKC-epsilon, Ephrin-A receptor 2, p90Rsk, IP3 receptor, NGF, AP-1, MAPKAPK2, Calmodulin, MAGI-2, PKC-lambda/iota, N-Ras, SNX26 (TCGAP), MAP2K5 (MEK5), CREB1, MEK1/2, PLAUR (uPAR), MMP-10, SP1, RGS2, PVR, SHB, RUSC1 (NESCA), Stromelysin-1, RIT, Elk-1, SRF, FosB
**25**	Immune response_IL-3 signaling via ERK and PI3K	8.025E-11	4.876E-09	GAB2, ITGB1, JAK2, SGK3, cPKC (conventional), PI3K cat class IA, Calcineurin A (catalytic), GRB2, c-Fos, PREL1, H-Ras, ERK1/2, CrkL, EGR1, alpha-5/beta-1 integrin, C3G, GATA-2, PDE4, PKC-epsilon, p90Rsk, PI3K cat class IA (p110-delta), Paxillin, Rac1, IP3 receptor, BAD, Talin, p70 S6 kinases, GLUT1, PI3K reg class IA (p85), GSK3 beta,

				Calmodulin, FLII, MDM2, FAK1, GSK3 alpha/beta, CREB1, Bcl-XL, MEK1/2, STAT5, CDC42, E4BP4, AKT(PKB), Semaphorin 7A, PDK (PDPK1), p27KIP1, Mcl-1, PUMA, Elk-1, LPCAT2, mTOR
**26**	Role of stellate cells in progression of pancreatic cancer	1.148E-10	6.706E-09	COL1A1, GRO-2, MEK1(MAP2K1), PI3K cat class IA, RECK, MMP-13, GRB2, c-Fos, IL-8, TGF-beta receptor type II, EGFR, H-Ras, c-Jun, ERK1/2, HB-EGF, ID1, alpha-5/beta-1 integrin, PDGF-R-alpha, Fibronectin, NFKBIA, Collagen III, NGF, PI3K reg class IA (p85), MEK2(MAP2K2), MMP-2, FAK1, IL-6, COL1A2, CTGF, AKT(PKB), PDGF receptor, PDK (PDPK1), Stromelysin-1, Collagen I, PDGF-R-beta
**27**	IL-1 beta- and Endothelin-1-induced fibroblast/ myofibroblast migration and extracellular matrix production in asthmatic airways	1.356E-10	7.629E-09	COL1A1, IL-1 alpha, MMP-1, c-Jun, ERK1/2, Versican, c-Jun/c-Fos, PDGF-R-alpha, Thrombospondin 1, Fibronectin, Collagen III, IL-1 beta, AP-1, EDNRB, Decorin, HAS2, MMP-2, IL-1RI, COL1A2, NF-kB, CTGF, ABCC5, Stromelysin-1, PAI1, EDNRA, Collagen I, PDGF-R-beta
**28**	Protein folding and maturation_Amyloid precursor protein processing (schema)	1.525E-10	8.272E-09	Caspase-6, APP-P3, alphaAPPs, BACE1, APP-C99, etaAPP alpha, Caspase-9, APP, APP-C31, APP-CTF delta-short, APP-CTF delta-long, betaAPPs, ADAM9, deltaAPPs-80kD, etaAPP beta, APP-CTF theta, Caspase-3, BACE2, APP-C83 (CTF), APP-NCas, Amyloid beta 40, Legumain, APP-CTF eta, thetaAPPs, Amyloid beta, etaAPPs, ADAM17, APP-C59 (AICD), deltaAPPs-130kD, APP-Jcasp, Amyloid beta 42
**29**	Cell cycle_Role of Cul1/Rbx1 E3 ligase in cell cycle regulation	1.940E-10	9.821E-09	E2F1, beta-TrCP, NEDD8, p21, SKP1, Cul1/Rbx1 E3 ligase, CDK1 (p34), CDK2, Cullin 1, Ubiquitin, Chk1, UBE1, CKS1, CDK4, p130, Skp2/TrCP/FBXW, RING-box protein 1, SKP2, Cdt1, Emi1, p27KIP1, PLK1
**30**	Abnormalities in cell cycle in small cell lung cancer (SCLC)	1.940E-10	9.821E-09	p14ARF, Histone H3, E2F1, p21, E2F3, CDK1 (p34), Cyclin E2, CDK2, CDK6, PCNA, E2F2, CKS1, Miz-1, CDK4, MDM2, Cyclin A, SKP2, Max, Cyclin B1, p16INK4, p27KIP1, c-Myc
**31**	Cell cycle_Influence of Ras and Rho proteins on G1/S Transition	2.075E-10	1.017E-08	MEK1(MAP2K1), PI3K cat class IA, ROCK2, E2F1, H-Ras, ERK1/2, alpha-5/beta-1 integrin, p21, NFKBIA, Rac1, CDK2, CDK6, GSK3 beta, p70 S6 kinase1, PI3K reg class IA, CDK4, Cyclin A2, ATF-2, Tob1, MRLC, MEK2(MAP2K2), MDM2, FAK1, RGL2, CDC42, SKP2, LIMK2, AKT(PKB), PDK (PDPK1), RalA, p27KIP1, c-Myc
**32**	Cell cycle_DNA replication: elongation and termination	2.586E-10	1.228E-08	RFC4, POLE1, MCM3, POLE4, MCM5, MCM4, GINS complex, VCP, FEN1, POLD cat (p125), DNA ligase I, RNaseh2b, CDK1 (p34), CDK2, Ubiquitin, PCNA, Chk1,

				UBE1, MCM7, RFC3, CHTF18, SHPRH, Ribonuclease H1, RFC1, RFC2, Cyclin A, UBE2G2, TIM, MCM2, POLD reg (p50), RNASEH2A, POLE3 (YBL1), LRR-1, UBE2D2, SMARCA3, CDC45L
**33**	Regulation and signaling of HGF receptor (Met) and MSP receptor (RON) in lung cancer	3.746E-10	1.724E-08	PI3K cat class IA, NCK1, GRB2, c-Fos, K-RAS, HIF1A, C/EBPbeta, H-Ras, c-Jun, ERK1/2, CrkL, Beta-catenin, c-Jun/c-Fos, VEGF-A, MSP, Alpha adducin, Thrombospondin 1, p90Rsk, Paxillin, Rac1, ATF-2/c-Jun, CDK1 (p34), COX-2 (PTGS2), IL-1 beta, PI3K reg class IA (p85), AP-1, GSK3 beta, p70 S6 kinase1, PKC-alpha, ATF-2, MMP-2, FAK1, CREB1, IL-6, CDC42, AKT(PKB), SP1, PDK (PDPK1), Gamma adducin
**34**	Development_Gastrin in cell growth and proliferation	4.009E-10	1.763E-08	MEK1(MAP2K1), JAK2, PI3K cat class IA, PI3K reg class IA (p85-alpha), GRB2, c-Fos, IRS-1, EGFR, H-Ras, TCF7L2 (TCF4), c-Jun, ERK1/2, HB-EGF, Beta-catenin, c-Jun/c-Fos, G-protein alpha-q/11, PKC-epsilon, p90Rsk, IP3 receptor, PRKD1, PI3K reg class IA (p85), AP-1, p70 S6 kinase1, PKC-alpha, MEK2(MAP2K2), FAK1, CREB1, G-protein alpha-q, JNK(MAPK8-10), ERK2 (MAPK1), PDK (PDPK1), p70 S6 kinase2, Stromelysin-1, c-Myc, ERK1 (MAPK3)
**35**	Development_Positive regulation of WNT/Beta-catenin signaling in the cytoplasm	4.063E-10	1.763E-08	Casein kinase II, alpha chains, GSKIP, TBL1X, ITGB1, Bcl-9, BIG1, GRB2, SIAH2, IRS-2, HIPK2, IRS-1, Tcf(Lef), PP1-cat, c-Jun, 14-3-3, Beta-catenin, TGIF, PP2A catalytic, RNF220, Dsh, Rac1, CDK1 (p34), COX-2 (PTGS2), Trabid, Miz-1, SIAH1, USP25, PR130, WNT, USP7, FAK1, GSK3 alpha/beta, TBLR1, JNK(MAPK8-10), ERK2 (MAPK1), AKT(PKB), Axin, MITF, Frizzled, DACT1
**36**	Development_Role of IL-8 in angiogenesis	4.667E-10	1.969E-08	MEK1(MAP2K1), JAK2, PI3K cat class IA, PI3K reg class IA (p85-alpha), SREBP1 (nuclear), c-Fos, IL-8, EGFR, G-protein alpha-i family, ERK1/2, HB-EGF, VEGF-A, SREBP2 (nuclear), FASN, SREBP1 (Golgi membrane), SREBP2 precursor, Cathepsin B, Paxillin, Rac1, Ubiquitin, SREBP1 precursor, I-kB, PI3K reg class IA (p85), LDLR, MALT1, HMDH, SREBP2 (Golgi membrane), MMP-14, FAK1, IKK-gamma, G-protein beta/gamma, Bcl-10, NF-kB, AKT(PKB), PDK (PDPK1), Elk-1
**37**	IGF signaling in lung cancer	4.941E-10	2.029E-08	MEK1(MAP2K1), PI3K cat class IA, ERK5 (MAPK7), RHEB2, GRB2, IRS-2, IRS-1, H-Ras, ERK1/2, VEGF-A, MSK1/2 (RPS6KA5/4), p90Rsk, IBP3, BAD, p70 S6 kinase1, Survivin, PI3K reg class IA, IGF-2, ATM, MAP2K5 (MEK5), MEK2(MAP2K2), MMP-2, CREB1, Bcl-XL, AKT(PKB), PDK (PDPK1), IBP, mTOR

**38**	Immune response_Gastrin in inflammatory response	5.293E-10	2.116E-08	GRO-2, MEK1(MAP2K1), PI3K cat class IA, ERK5 (MAPK7), GRB2, c-Fos, IRS-1, IL-8, EGFR, H-Ras, c-Jun, HB-EGF, MEF2, G-protein alpha-q/11, PKC-epsilon, IP3 receptor, ATF-2/c-Jun, COX-2 (PTGS2), I-kB, MEF2D, PI3K reg class IA (p85), PKC-alpha, ATF-2, IKK-beta, MAP2K5 (MEK5), MEK2(MAP2K2), FAK1, CREB1, G-protein alpha-q, JNK(MAPK8-10), ERK2 (MAPK1), AKT(PKB), PDK (PDPK1), Stromelysin-1, ERK1 (MAPK3), Elk-1, TAK1(MAP3K7)
**39**	IGF family signaling in colorectal cancer	6.211E-10	2.373E-08	PI3K reg class IA (p85-alpha), GRB2, c-Fos, IRS-2, HIF1A, IRS-1, IL-8, E2F1, H-Ras, c-Jun, ERK1/2, Beta-catenin, VEGF-A, IBP3, COX-2 (PTGS2), GIPC, I-kB, GSK3 beta, p70 S6 kinase1, MAFG, PTEN, IGF-2, GSK3 alpha/beta, Bcl-XL, MNK2(GPRK7), MEK1/2, NF-kB, ERK2 (MAPK1), AKT(PKB), ERK1 (MAPK3), IBP, MAT2A, mTOR, FosB
**40**	Th2 cytokine- and TNF-alpha-induced profibrotic response in asthmatic airway fibroblasts/ myofibroblasts	6.249E-10	2.373E-08	COL1A1, GRB2, FAN, MMP-1, ETS1, IL-4R type II, c-Jun, ERK1/2, BDKRB1, c-Jun/c-Fos, PDGF-R-alpha, Collagen III, COX-2 (PTGS2), IL-1 beta, NGF, COX-1 (PTGS1), AP-1, Decorin, Tenascin-C, HAS2, MMP-2, MEK1/2, IL13RA2, JNK(MAPK8-10), COL1A2, NF-kB, ABCC5, AKT(PKB), SP1, Collagen I, TNF-alpha
**41**	Suppression of p53 signaling in multiple myeloma	6.412E-10	2.376E-08	DR5(TNFRSF10B), p14ARF, ATF-3, Apaf-1, c-Jun, VEGF-A, Caspase-9, UBCH8, p21, Bak, Cytochrome c, Bax, Survivin, PCAF, Caspase-3, FasR(CD95), MDM2, IL-6, GADD45 alpha, NOXA, DR4(TNFRSF10A), c-Myc, Mcl-1, PUMA, PSMD4
**42**	Signal transduction_S1P2 receptor activation signaling	6.855E-10	2.479E-08	JAK2, G-protein alpha-12 family, PI3K cat class IA, c-Fos, G-protein alpha-i family, H-Ras, beta-TrCP, c-Jun, FOXM1, ERK1/2, HB-EGF, SMAD9 (SMAD8), Beta-catenin, EGR1, c-Jun/c-Fos, G-protein alpha-i2, NFKBIA, Paxillin, IP3 receptor, G-protein alpha-13, COX-2 (PTGS2), ROCK, PI3K reg class IA (p85), AP-1, GSK3 beta, GRO-1, Survivin, RUNX2, PLC-beta, IKK-beta, MRLC, LIF, FAK1, CREB1, Transgelin, MEK1/2, G-protein beta/gamma, Actin cytoskeletal, G-protein alpha-q, JNK(MAPK8-10), NF-kB, LIMK2, MKL1, Osteoprotegerin, AKT(PKB), PDK (PDPK1), PPAR-gamma, SRF, Cofilin, TNF-alpha
**43**	Cytoskeleton remodeling_PDGF signaling via calcium and Rho GTPases	7.411E-10	2.618E-08	SLC31A1, ABL2, AKT1, Lysyl oxidase, NCK1, PLD1, F-Actin, DOCK1, Beta-catenin, Guanylate cyclase A (NPR1), EBP50, PDGF-R-alpha, WASF subunit, WASF3 (WAVE3), Dynamin-2, PKC-epsilon, Paxillin, Rac1, IP3 receptor, PI3K

				reg class IA (p85), Calmodulin, PKC, Vinculin, PKC-alpha, VAV-2, FAK1, c-Abl, PDGF-C, Cortactin, CDC42, Actin cytoskeletal, Fyn, PDGFR-ab, ATOX1, PDGF receptor, ALPHA-PIX, WASF2, PDGF-D, ARPC2, WaspIP, PDGF-R-beta, ATP7A
**44**	Development_Negative regulation of WNT/Beta-catenin signaling in the nucleus	7.675E-10	2.650E-08	KDM2, TBL1X, Casein kinase I delta, AKT1, TRRAP, Calcineurin A (catalytic), NARF, RUNX3, HBP1, Tcf(Lef), E2F1, TCF7L2 (TCF4), 14-3-3, Jade-1, Beta-catenin, VEGF-A, TRIM33, BCL9/B9L, GLI-3R, PC1-CTT, TLE, CBP/P300, Dsh, Cul1/Rbx1 E3 ligase, NLK, WNT5A, GSK3 beta, WNT, LATS2, CHIBBY, TBLR1, HDAC1, Plakoglobin, PJA2, HIC5, RUVBL2, PPAR-gamma, Axin, TAK1(MAP3K7), SOX9, CDX1, Frizzled, Histone H1, DACT1
**45**	Apoptotic pathways and resistance to apoptosis in lung cancer cells	7.905E-10	2.668E-08	MEK1(MAP2K1), c-IAP2, DR5(TNFRSF10B), Apaf-1, ERK1/2, CYLD, TRADD, Caspase-9, Cytochrome c, FADD, PKC-epsilon, Bax, Survivin, PKC-alpha, c-IAP1, PEA15, tBid, Caspase-3, RIPK1, FasR(CD95), MEK2(MAP2K2), Bcl-XL, HDAC1, NF-kB, DR4(TNFRSF10A), Caspase-1, p70 S6 kinase2, p27KIP1, Mcl-1, Smac/Diablo, Bid, TNF-alpha
**46**	TGF-beta signaling via kinase cascades in breast cancer	9.612E-10	3.174E-08	MEK1(MAP2K1), ITGB1, MMP-13, GRB2, c-Fos, NF-kB1 (p50), ADAM12, Neuregulin 1, ITGB3, IL-8, TGF-beta receptor type II, EGFR, c-Jun, ERK1/2, NFKBIA, Rac1, ATF-2/c-Jun, COX-2 (PTGS2), Ubiquitin, AP-1, Survivin, ATF-2, IKK-beta, TAB1, MMP-2, FAK1, JNK(MAPK8-10), TWIST1, AKT(PKB), ADAM17, PAI1, TAK1(MAP3K7), TNF-alpha
**47**	Neurophysiological process_Melatonin signaling in the nervous system	1.000E-09	3.232E-08	Heme oxygenase 1, GnRH1, cPKC (conventional), PI3K cat class IA, MUPP1, ROR-alpha, C/EBPbeta, G-protein alpha-i family, ERK1/2, Beta-catenin, CRMP2, G-protein alpha-i3, G-protein alpha-q/11, G-protein alpha-i2, p90Rsk, IP3 receptor, BAD, 14-3-3 beta/alpha, ROCK, GLUT1, PI3K reg class IA (p85), Bax, Sirtuin1, GSK3 beta, Calmodulin, Survivin, PKC, PLC-beta, PKC-alpha, Adenylate cyclase, CREB1, MEK1/2, G-protein beta/gamma, NF-kB, AKT(PKB), PDK (PDPK1), NRF2, BDNF
**48**	Proteolysis_Ubiquitination pathway	1.291E-09	4.086E-08	c-IAP2, WWP2, MARCH4, SIAH2, UBE2Q2, UBE2H, UBE3A (E6-AP), FBXW7, MARCH2, UBCH7, GCN5, CBL-B, HERC4, UBCH8, UBE2S, Cullin 4, Cullin 1, Ubiquitin, RNF4, UBE2D4, UBE2E2, UBE1, PCAF, UBCH6, FLJ13855, UBE2D3, Itch, AMFR, MDM2,

				UBE2G2, RING-box protein 1, Mahogunin, HIP-2, UBE2D2, UBC6
**49**	Ligand-independent activation of Androgen receptor in Prostate Cancer	1.439E-09	4.371E-08	MEK1(MAP2K1), STAT5A, JAK2, PI3K cat class IA, GRB2, K-RAS, NCOA3 (pCIP/SRC3), IRS-1, Neuregulin 1, Tcf(Lef), EGFR, H-Ras, Beta-catenin, PP2A regulatory, PP2A catalytic, DDX5, STAT5B, IBP3, Kallikrein 3 (PSA), GSK3 beta, PI3K reg class IA, FRS2beta, N-Ras, MEK2(MAP2K2), MDM2, c-Abl, Bcl-XL, NCOA1 (SRC1), HDAC1, ERK2 (MAPK1), AKT(PKB), SRD5A1, PDK (PDPK1), c-Myc, ERK1 (MAPK3), Frizzled
**50**	Development_Thrombopoietin signaling via ERK1/2 and PI3K	1.439E-09	4.371E-08	GAB2, PDK1, JAK2, PI3K cat class IA, Cyclin D3, GRB2, c-Fos, ITGA2B, IRS-2, HIF1A, PP1-cat, H-Ras, ERK1/2, CrkL, EGR1, HOXA9, VEGF-A, p21, USF1, C3G, AML1 (RUNX1), PI3K reg class IA (p85), GSK3 beta, p70 S6 kinase1, MEIS1, c-MPL, CREB1, Bcl-XL, MEK1/2, AKT(PKB), SP1, FLI1, PDK (PDPK1), p27KIP1, c-Myc, Elk-1

Supplementary Table 4: MetaCore pathway enrichment of specific DEGs in chronic chromium exposure. Top 50 Pathway Maps significantly enriched among DEGs in GSE24025 (chromium-exposed vs. control; DEGs defined at $|FC| \geq 1.2$ and BH-adjusted $p < 0.05$).

#	Maps	pValue	Min FDR	Network Objects from Active Data
**1**	Stellate cells activation and liver fibrosis	2.456E-10	3.365E-07	IL-1RI, Biglycan, I-kB, TGF-beta 1, Cyclin D1, IRAK1/2, SOS, PDGF-B, SMAD3, AKT(PKB), Tcf(Lef), SARA, TGF-beta receptor type II, Frizzled, IL1RAP, Smoothened, KLF6, MMP-2
**2**	Cell cycle_Regulation of G1/S transition (part 1)	2.117E-07	1.450E-04	TGF-beta 1, Cyclin A, Skp2/TrCP/FBXW, Cyclin D1, PP2A catalytic, SMAD3, JunB, p16INK4, SARA, TGF-beta receptor type II, Cyclin D
**3**	SHH signaling in colorectal cancer	2.682E-06	9.391E-04	FOXC2, HIP, GLI-3R, SNAIL1, GLI-3, IMP1(ZBP1), TCF7L2 (TCF4), Frizzled, Smoothened
**4**	Stimulation of TGF-beta signaling in lung cancer	2.742E-06	9.391E-04	I-kB, TGF-beta 1, TGF-beta, Tropomyosin-1, SNAIL1, SMAD3, AKT(PKB), Furin, TGF-beta receptor type II, IRAK1, MMP-2
**5**	TGF-beta-induced fibroblast/ myofibroblast migration and extracellular matrix production in asthmatic airways	4.476E-06	1.226E-03	Biglycan, TGF-beta 1, AP-1, COL4A1, SMAD3, TIMP3, ABCC5, AKT(PKB), COL5A1, Collagen IV, TGF-beta receptor type II, MMP-2
**6**	Development_EGFR signaling	6.033E-06	1.377E-03	HB-EGF, Cyclin D1, AP-1, SOS, SOS2, AKT(PKB), PI3K reg class IA, GAB1, c-Cbl, Epiregulin, Mcl-1, p90Rsk, STAT1, MMP-2

**7**	Inhibition of TGF-beta 1 signaling in early colorectal cancer	8.247E-06	1.449E-03	TGF-beta 1, SMAD3, SMAD6, AKT(PKB), PI3K reg class IA, SMAD7, TGF-beta receptor type II, MMP-2
**8**	DNA damage_ATM/ATR regulation of G2/M checkpoint: nuclear signaling	8.464E-06	1.449E-03	CDC18L (CDC6), Cyclin A, Chk1, HDAC6, Cyclin B, Cyclin B2, PCBP-4 (mcg10), Mcl-1, p53BP1, CEP164
**9**	Beta-catenin-dependent transcription regulation in colorectal cancer	1.043E-05	1.588E-03	Cyclin D1, LAMC2, LAMC2 (80kDa), LAMC2 (100kDa), Claudin-1, SOX9, TCF7L2 (TCF4), L1CAM, Fascin
**10**	Cell cycle_Role of APC in cell cycle regulation	3.261E-05	4.468E-03	MAD2b, CDC18L (CDC6), Cyclin A, Aurora-B, SKP2, Cyclin B, ORC1L, Aurora-A
**11**	Normal and pathological TGF-beta-mediated regulation of cell proliferation	4.153E-05	5.172E-03	TGF-beta 1, Cyclin D1, SOS, PDGF-B, SMAD3, ITGAV, SARA, TGF-beta receptor type II
**12**	IL-6 signaling in breast cancer cells	4.777E-05	5.454E-03	gp130, Cyclin D1, SNAIL1, SOS, AKT(PKB), PI3K reg class IA, GAB1, Mcl-1, Fascin, STAT1
**13**	Signal transduction_Angiotensin II/ AGTR1 signaling via p38, ERK and PI3K	5.327E-05	5.614E-03	AGTR1, HB-EGF, PDGF-C, MEF2A, Cyclin D1, SOS, PDGF-B, AKT(PKB), TLR4, p90Rsk, MAP2K5 (MEK5), ATP7A, MMP-2, GCN5
**14**	Signal transduction_Production and activation of TGF-beta in airway smooth muscle cells	6.549E-05	6.409E-03	AGTR1, TGF-beta 1, TGF-beta, AP-1, TGF-beta receptor type II, PAR2, TLR4, IRAK1
**15**	Regulation of metabolism_GLP-1 signaling in beta cells	7.917E-05	7.231E-03	AMPK alpha subunit, I-kB, Cyclin D1, Amylin, AKT(PKB), PDHA (somatic), GCL cat, IBP1, PDZ-GEF1, p90Rsk, TCF7L2 (TCF4), ATP6AP2, 14-3-3
**16**	TGF-beta signaling via SMADs in breast cancer	1.011E-04	8.575E-03	TGF-beta 1, PTHrP, SNAIL1, SMAD3, NOX4, JunB, TGF-beta receptor type II, Fascin, TBX2
**17**	G-protein signaling_RhoB activation	1.064E-04	8.575E-03	TGF-beta, Chk1, PDGF-B, PIAS1, SMAD3, AKT(PKB), GCR, RhoB, TGF-beta receptor type II, TLR4
**18**	Development_Oligodendrocyte differentiation (general schema)	1.221E-04	9.297E-03	HB-EGF, SMAD1, CD82, PDGF-B, SMAD6, BMP receptor 2, CNP1, SMAD7
**19**	Abnormalities in cell cycle in small cell lung cancer (SCLC)	1.305E-04	9.408E-03	Cyclin A, Cyclin D1, p14ARF, Aurora-B, SKP2, Histone H3, p16INK4
**20**	Activation of TGF-beta signaling in advanced colorectal cancer	1.642E-04	1.113E-02	TGF-beta 1, Cyclin D1, SNAIL1, SMAD3, AKT(PKB), PI3K reg class IA, TGF-beta receptor type II
**21**	WNT signaling in hepatocellular carcinoma (HCC)	1.788E-04	1.113E-02	Prickle-1, Cyclin D1, p14ARF, FZD6, Tcf(Lef), TCF7L2 (TCF4), Frizzled, DKK1

**22**	IL-6 signaling in Prostate Cancer	1.788E-04	1.113E-02	gp130, Cyclin D1, AP-1, SOS, AKT(PKB), PI3K reg class IA, GAB1, Mcl-1
**23**	Development_Regulation of epithelial-to-mesenchymal transition (EMT)	2.170E-04	1.293E-02	IL-1RI, TGF-beta 1, Tropomyosin-1, SNAIL1, PDGF-B, TCF8, Claudin-1, TGF-beta receptor type II, Frizzled, MMP-2
**24**	Stem cells_Cooperation between Hedgehog, IGF-2 and HGF signaling pathways in medulloblastoma stem cells	2.530E-04	1.345E-02	Cyclin D1, AKT(PKB), PI3K reg class IA, GAB1, Tcf(Lef), TCF7L2 (TCF4), Smoothened
**25**	Cell cycle_Role of Nek in cell cycle regulation	2.530E-04	1.345E-02	NEK1, Tubulin gamma, Tubulin alpha, Histone H3, PI3K reg class IA, Aurora-A, Insulin receptor
**26**	Signal transduction_WNT/Beta-catenin signaling in tissue homeostasis	2.553E-04	1.345E-02	Cyclin D1, MENA, SNAIL1, Cyclin A2, Tcf(Lef), TCF7L2 (TCF4), Frizzled, MMP-2
**27**	Development_Non-canonical TGF-beta signaling via PI3K, RhoA, and ROS	2.825E-04	1.369E-02	RhoA-related, TGF-beta 1, TGF-beta, Tropomyosin-1, PP2A catalytic, SMAD3, AKT(PKB), NOX4, RhoB, TGF-beta receptor type II
**28**	Signal transduction_Activin A signaling	2.825E-04	1.369E-02	ActRIIA, FSRP, Cyclin D1, AP-1, MAFbx, SMAD3, AKT(PKB), PI3K reg class IA, p16INK4, SOX9
**29**	TGF-beta 1-mediated induction of EMT in normal and asthmatic airway epithelium	3.026E-04	1.369E-02	TGF-beta 1, AP-1, SNAIL1, SMAD3, SMAD7, TGF-beta receptor type II, ZO-1, MMP-2
**30**	Cell cycle_Senescence activation pathways	3.060E-04	1.369E-02	AMPK alpha subunit, TGF-beta 1, Chk1, Cyclin D1, p14ARF, SMAD3, AKT(PKB), p16INK4, p90Rsk
**31**	Role of metalloproteases and heparanase in progression of pancreatic cancer	3.099E-04	1.369E-02	HB-EGF, SOS, Heparanase 1, Collagen IV, Alpha 1-antitrypsin, ZO-1, MMP-2
**32**	Hedgehog signaling in breast cancer	3.250E-04	1.392E-02	Cyclin D1, PTHrP, CD24, XIAP, Smoothened, MMP-2
**33**	Neurogenesis_NGF/ TrkA MAPK-mediated signaling	3.424E-04	1.422E-02	KIDINS220, HB-EGF, SHPS-1, Cyclin D1, AP-1, SOS, PP2A catalytic, JunB, RGS2, SHB, PDZ-GEF1, p90Rsk, MAP2K5 (MEK5)
**34**	Signal transduction_AKT(PKB) activation	4.112E-04	1.657E-02	IL-1RI, Rictor, Cyclin A, SKP2, PIAS1, AKT(PKB), Insulin receptor, NEDD4, PDGF-BB, AKT3
**35**	Higher ESR1 / ESR2 ratio in breast cancer	4.544E-04	1.719E-02	p130, ID1, Cyclin D1, SKP2, PDGF-B, AKT(PKB), Cyclin A2
**36**	Development_Canonical TGF-beta signaling	4.637E-04	1.719E-02	TGF-beta 1, TGF-beta, AP-1, SNAIL1, SMAD3, SARA, SMAD7, TCF8, Claudin-1, TGF-beta receptor type II

**37**	EGFR signaling in head and neck squamous cell carcinoma (HNSCC)	4.644E-04	1.719E-02	HB-EGF, CDC18L (CDC6), Cyclin D1, AKT(PKB), PI3K reg class IA, Semaphorin 7A, c-Cbl, FUT4, Epiregulin
**38**	Prolactin/ ERK signaling in breast cancer	5.445E-04	1.913E-02	GSTM1, Cyclin D1, SOS, AKT(PKB), JunB, NEK3, MMP-2
**39**	Cell cycle_The metaphase checkpoint	5.445E-04	1.913E-02	MAD2b, Rod, Aurora-B, CENP-C, ZW10, CENP-E, Aurora-A
**40**	Development_PtdIns(3,4,5)P3 signaling in cardiac myocytes	5.685E-04	1.947E-02	SOS, AKT(PKB), PI3K reg class IA, GAB1, Insulin receptor, p90Rsk, Cyclin D, 14-3-3
**41**	Activation of TGF-beta signaling in pancreatic cancer	6.440E-04	2.104E-02	TGF-beta 1, SOS, PDGF-B, SMAD3, TGF-beta receptor type II, L1CAM
**42**	G-protein signaling_H-RAS regulation pathway	6.482E-04	2.104E-02	GDNF, p120GAP, ICMT, SOS, PDGF-B, PDZ-GEF1, GFRalpha1
**43**	Induction of fibrosis in systemic sclerosis	6.605E-04	2.104E-02	IL-1RI, TGF-beta 1, SMAD1, SMAD3, LAP beta 1, COL5A1, SMAD7, TGF-beta receptor type II, TLR4, Frizzled, DKK1
**44**	Role of stellate cells in progression of pancreatic cancer	6.846E-04	2.132E-02	HB-EGF, ID1, TGF-beta 1, SOS, PDGF-B, SMAD3, AKT(PKB), TGF-beta receptor type II, MMP-2
**45**	Role of TGF-beta 1 in fibrosis development after myocardial infarction	7.670E-04	2.308E-02	AGTR1, Biglycan, TGF-beta 1, SMAD3, SMAD7, TGF-beta receptor type II, MMP-2
**46**	Cell cycle_Regulation of G1/S transition (part 2)	7.917E-04	2.308E-02	p130, Cyclin A, Cyclin D1, AKT(PKB), Cyclin A2, Cyclin D
**47**	Development_Role of CNTF and LIF in regulation of oligodendrocyte development	7.917E-04	2.308E-02	gp130, Annexin V, AKT(PKB), PI3K reg class IA, STAT1, 14-3-3
**48**	TGF-beta 1-induced transactivation of membrane receptors signaling in hepatocellular carcinoma (HCC)	8.718E-04	2.488E-02	TGF-beta 1, Cyclin A, TGF-beta, Cyclin D1, SNAIL1, AKT(PKB), PI3K reg class IA, TGF-beta receptor type II
**49**	Cell cycle_Role of Cul1/Rbx1 E3 ligase in cell cycle regulation	9.642E-04	2.492E-02	p130, Skp2/TrCP/FBXW, Chk1, Cyclin D1, SKP2, SMAD3
**50**	Suppression of TGF-beta signaling in pancreatic cancer	9.642E-04	2.492E-02	Biglycan, TGF-beta 1, SMAD3, SMAD6, SMAD7, TGF-beta receptor type II
